# Greenness and Pricing Decisions of Cooperative Supply Chains Considering Altruistic Preferences

**DOI:** 10.3390/ijerph16010051

**Published:** 2018-12-26

**Authors:** Hui Huang, Juan Zhang, Xuan Ren, Xiang Zhou

**Affiliations:** School of Management, Northwestern Polytechnical University, Xi’an 710129, China; hhnwpu@nwpu.edu.cn (H.H.); renxuan@mail.nwpu.edu.cn (X.R.); zhouxiang1996@mail.nwpu.edu.cn (X.Z.)

**Keywords:** green supply chain, altruistic preference, pricing, greenness

## Abstract

With the development of the economy and science technology, global resource shortages and environmental pressures have become the focus of attention. More and more consumers tend to buy non-polluting and environmentally friendly green products, and many manufacturers and retailers are beginning to produce or sell green products to enhance their competitive advantage in the market. Considering the green preference attributes of consumers, the altruistic preference is introduced into the supply chain, and we establish four models: two cooperative manufacturers and one retailer are completely self-interested, one retailer has altruistic preference, two cooperative manufacturers have altruistic preferences, and two cooperative manufacturers and one retailer have altruistic preferences. We address the optimal greenness and pricing decisions of supply chain members, and analyze the impact of altruistic preferences on supply chain decision-making and profits. The results show that the altruistic preference coefficient can significantly affect the decision-making and the profits of supply chain members, and when two manufacturers and one retailer consider altruistic preferences, the altruistic preference coefficients adopted by the three parties are in the certain ranges, the supply chain members’ altruistic preference coefficients can increase the whole supply chain profit. Through analysis, in the three cases where the retailer has altruistic preferences, two manufacturers have altruistic preferences, and two manufacturers and one retailer have altruistic preferences, two manufacturers should adopt higher altruistic preference coefficients, and the retailer should adopt a lower altruistic preference coefficient, and the product greenness under the three altruistic preferences is higher than the product greenness when there is no altruistic preference.

## 1. Introduction

In recent years, resource shortages and environmental pollution have prompted enterprises to engage in the research and development of green products. Meanwhile, consumers’ preference for switching from ordinary products to green products has become more and more evident and frequent. In order to satisfy environmental pressure and consumers’ increasing pursuit of daily life quality, more economical and environmentally friendly goods, like organic food and green vegetables in supermarkets, are produced by manufacturers. As a result, the competition among enterprises has evolved from a single price competition to a price-green competition. Haier adopts green core technology to accelerate the upgrading of home appliance exports. By developing an efficient power strategy, Bavarian Motor Work Brilliance achieves the goal of increasing power and reducing fuel consumption to reduce environmental impact. However, the research and development of green products requires high technological level and cost, which forces many companies to develop products through cooperation. Wholesale prices would be higher under a cooperative relationship because enterprises will inevitably attract retailers through price strategies to obtain more orders under a competitive relationship. Toyota Motors and BMW have cooperated in the research and development of automobile products. By cooperating, the two brands of water heaters, “Shenzhou” and “Wanjiale” quickly entered thousands of households, bringing huge profits to the two companies. It can be seen that if enterprises want to achieve long-term development, they must not only have competition, but also know how to cooperate. In practice, we could observe that manufacturers and retailers, in order to improve their interests or image, may consider altruistic preferences when green products are produced or sold, which motivates our research. In addition, through our research, we could find that manufacturers and retailers adopt altruistic preferences that can increase the whole supply chain profit, which attests the value of our research.

In addition, the society has become more and more concerned about environmental protection. In order to achieve sustainable development of the environment, different countries have introduced relevant policies to encourage the development of economic and environmental protection enterprises, as well as impose penalties on enterprises that cause serious environmental pollution. The Copenhagen World Climate Conference was held at the Bella Center in Copenhagen from 7 to 18 December 2009. In the face of environmental pressures, production with higher greenness was advocated to be the direction of current enterprise development. For example, Huawei implements an environmental strategy based on the concept of “circular economy”, continuously improving the efficiency of resource and energy use. Haier produces products with strategic objectives of green design, green manufacturing, green management and green recycling.

In this paper, considering the phenomenon of altruistic preferences existing in enterprises, we explore the pricing and greenness decision-making of products in the production and sales of products by two cooperative manufacturers and a retail chain. Firstly, we analyze the types of altruistic preferences that may exist between two manufacturing and a retailer. Secondly, we analyze the optimal wholesale price and retail price for two manufacturers and a retailer in product development and sales, and the decision of the optimal green level. Finally, from the perspective of the optimal total profit of the supply chain, we analyze how to choose the altruistic preference level when obtaining the optimal pricing and greenness decision.

We find that two manufacturers and one retailer have altruistic preferences that not only increase product greenness, but also increase the whole supply chain profit. From the perspective of the whole supply chain profit, when two manufacturers and the retailer have altruistic preferences at the same time, if two manufacturers adopt higher altruistic preference coefficients and the retailer adopts lower altruistic preference coefficient, the whole supply chain profit is higher.

The reminder of this paper is organized as follows: Section 2 summarizes the related literature. Section 3 describes the problem and presents further assumptions and notations. Section 4 analyzes the optimal pricing and greenness with a two manufacturer Stackelberg game and obtains the profits and utilities of two manufacturers and one retailer, who are under different altruistic preference models. Section 5 verifies the correctness and efficiency of the models by numerical analysis. Conclusions are presented in Section 6.

## 2. Literature Review

### 2.1. Green Supply Chain

With the development of the economy and science technology, global resource shortages and environmental pressures have become the focus of attention. Fahimnia et al. [1] and Tseng et al. [2] found that consumers and companies are more concerned about environmental issues and the risks of climate change. Li et al. [3] and Sara et al. [4] found that more and more consumers tend to buy non-polluting and environmentally friendly green products, and many manufacturers and retailers are beginning to produce or sell green products to enhance their market competitive advantage. Against the background of people’s growing concerns about the sustainable use of natural resources, the continuous improvement of the ecological environment, the continuous improvement of the quality of life and the sustainable development of the economy, the green supply chain has become the strategic goal of industrial development in various countries. If enterprises want to achieve long-term development, take the initiative to undertake social and ecological environmental responsibility, and develop green products that are beneficial to the healthy development of society is the inevitable trend for enterprises to gain competitive advantages in the future. Kaur et al. [5] initially analyzed the barriers to the green supply chain in the Canadian electronics industry, helping decision makers, policy planners and organizational managers to address key players in the success of green supply chain practices. Zhang et al. [6] found that the combination of social regulation and green supply chain management practices has a positive and important role under a dynamic environmental condition. Bian et al. [7] reported that the environmental tax gets less stringent when the manufacturer distribution channel becomes more decentralized.

### 2.2. Supply Chain Pricing

There have been more studies on the issue of supply chain pricing. Ma et al. [8] studied the pricing of three-stage supply chains consisting of two competing manufacturers and one retailer. Jamali et al. [9] first investigated two alternative products to solve the problem of greenness and pricing of green products in competition with non-green products. Chen et al. [10] solved the optimal pricing problem of stakeholders by establishing three game theory models. Dey et al. [11] studied the retailer’s optimal retail pricing and purchasing decisions, as well as manufacturers’ wholesale pricing and product greening level decision in three-stage supply chain framework. Ma et al. [12] investigated closed-loop supply chains (CLSCs) under four reverse channel structures, and derived supply chain profitability and furnished the optimal marketing effort, collection rate and pricing decisions for the supply chain members. Qi et al. [13] studied a dual-channel supply chain coordination under a carbon cap-and-trade regulation, and obtained the optimal pricing decisions and corresponding profits in centralized and decentralized systems. Pu et al. [14] constructed four scenarios to uncover the influence of consumers’ preference on price and demand and the relationship between the influence coefficient of retailers’ promotional effort on consumers’ utility and retailer profits. Xu et al. [15] incorporated low-carbon development and environmental remediation into a resource-based supply chain coordination model and found that a centralized market can lead to improvement in total profit. Zhu et al. [16] found retailers and network recycling platforms would reduce the direct recovery prices to maintain their own profit when considering the impact of consumer bargaining behavior. Yang et al. [17] studied the pricing and carbon emission reduction rate of two competitive supply chain members under the cap-and-trade scheme. Zhu et al. [18] studied the impact of supply chain structure, product types and competition types on product greenness.

### 2.3. Product Greenness

Product greenness refers to the degree to which products are friendly to people and nature, such as the content of harmful substances, energy consumption, recyclability, etc., which have become an important factor affecting consumers’ purchasing decisions. Ghosh and Shah [19] studied the impact of channel structure on product greenness, pricing, and profitability of supply chain members, and proposed two pricing contracts to achieve supply chain coordination. Liu et al. [20] used game theory to study the impact of competition and consumer environmental awareness on the greenness and price decisions of supply chain member products, pointing out that the improvement of consumers’ environmental awareness can benefit high-environment-friendly enterprises. Zhang and Liu [21] studied the decision-making of three-level supply chain when market demand and product greenness were related. It was pointed out that revenue sharing mechanism, Shapley value method coordination mechanism and asymmetric Nash negotiation mechanism can improve supply chain performance under decentralized decision-making. Azevedo et al. [22] used the green index as a benchmark for assessing the lean, agility, adaptability and greenness of automotive companies and their corresponding supply chains.

### 2.4. Altruistic Preference

Traditional economics regards self-interest and competition as the soul of human beings, assuming that people are completely self-interested. However, a large number of psychological and behavioral economics studies have found that policy makers are not completely rationally self-interested. There are widespread behavioral preferences such as fairness, reciprocity, and altruism. These behavioral preferences have a wide-ranging impact on social and economic activities. Ge et al. [23] analyzed the impact of altruistic preferences on decentralized and concentrated supply chain performance. Liu et al. [24] Considering carbon tax regulation and consumers’ low-carbon preference, found that the manufacturer’s and the retailer’s fairness concerns decrease their product sustainability and low-carbon promotion level, together with the profits of the system and the manufacturer. Qin et al. [25] found that fairness preferences can improve supply chain profits and balance supply chain profit distribution. Nie et al. [26] found that manufacturers and retailers’ fairness concerns and distributional fairness concerns affect their decisions. Zhang et al. [27] studied the contractual preferences in a two-period supply chain under asymmetric information and examined whether the parties were willing to maintain the consistency of contract types during the two periods. Du et al. [28], when studying the relationship of an acrimonious supply chain and a harmonious supply chain decisions and reciprocal preferences, found that reciprocal preferences prevented wholesale price contracts from coordinating with a harmonized supply chain.

Altruistic preference means that when decision-makers choose actions, they not only consider the impact of actions on themselves, but also the consequences of actions on others. Members with altruistic preferences are willing to help others to promote social welfare. Zhang et al. [29] studied how to promote the upgrading of social altruistic behavior. Silk [30] explained the evolution of the extraordinary ability of large-scale cooperation and altruistic social preferences in human society. Urda et al. [31] pointed out that altruistic preferences not only affect decision utility functions, but also influence people’s decisions, emotions and behaviors. Olivella et al. [32] studied the altruistic preference between doctors and found that when paying more attention to personal reputation, doctors with lower altruism would imitate doctors with higher a preference.

In recent years, the academic community has gradually realized that it is impossible to fully explain the actual behavior of enterprises from the perspective of external incentives, and behavioral preferences must be studied as the intrinsic motivation of enterprise decision-making. In the manufacturer-led supply chain structure, when two manufacturers conduct their own research and development of green products, the cooperative relationship can obtain a higher wholesale price than the competitive one. Therefore, it is more practical to study the decision problem of cooperative supply chain members. This paper introduces the altruistic preference coefficients of supply chain members, considering the consumer’s green preference, and establishes four models: full self-interest, two manufacturers have altruistic preferences, the retailer has altruistic preference, and two manufacturers and the retailer have altruistic preferences, and analyzes the greenness and pricing decision of supply chain members in the four cases. Then we analyze the impact of altruistic preferences on wholesale prices, retail prices, greenness, supply chain member profits and utilities in different situations, and obtain the optimal decisions of manufacturers and the retailer with altruistic preferences. Finally, the correctness and efficiency of the models are verified by numerical examples.

## 3. Description and Assumptions

### 3.1. Model Description

**Figure 1:**
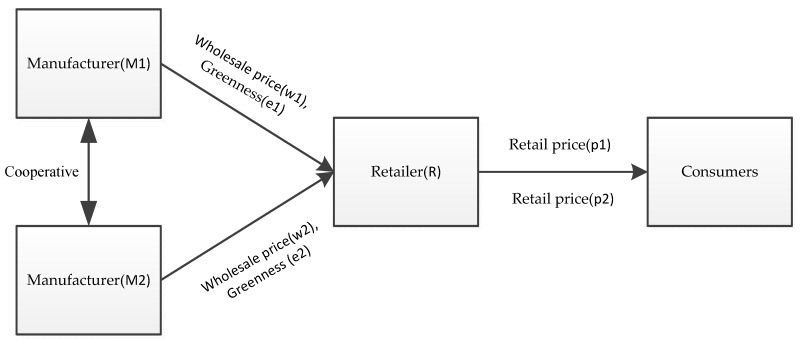
Manufacturers-cooperative three-stage supply chain.

We consider a three-stage supply chain with two manufacturers and one retailer (as shown in Figure 1). Two manufacturers produce green products, and there is a certain substitution between the two green products. Since the co-opetition relationship between the two manufacturers is cooperation, the manufacturers make decisions based on the principle of maximizing the profits of both. When the product greenness produced by the manufacturer *M_i_* is *e_i_*, the products are sold to the retailer *R* at unit wholesale price *w_i_*, and the retailer *R* sold them to consumers at unit price *p_i_*, where the product greenness and the retail prices of the products affect the consumers’ consumption.

With the advancement of the global greening process, consumers’ green preference trends have become more apparent. In order to gain a large market share, manufacturers will actively develop and produce green products. In the process of production, manufacturers need to invest a lot of money in research and production of green products. Therefore, two manufacturers will determine the wholesale prices according to the product greenness and the production costs. One retailer determines the retail prices based on the maximization of its own interest when the manufacturers have determined wholesale prices.

### 3.2. Notation

All the notations in supply chain decision models are shown in the Table 1.

*α*: The potential market demand quantity. Referring to the practice of Bian et al. [7], it is assumed that the demand of consumers for the type of products is certain in the market, when the number of consumers purchasing one of the products increases, the demand for another product of the same type will decrease.

*θ*: The cross-price sensitivity, which means that the demand for a product is sensitive to changes in the price of the substitutable product.

For the convenience of analysis, when the *M* or *R* is marked in the upper right corner of each symbol, it indicates that there is altruistic preference behavior, and *N* in the upper right corner represents self-interest behavior. For example, $w_{i}^{MMN}$ indicates the wholesale prices set by the manufacturers when the manufacturers have altruistic preferences and the retailer does not have altruistic preference, and $w_{i}^{MMR}$ indicates the wholesale prices set by the manufacturers when two manufacturers and the retailer have altruistic preferences.

### 3.3. Assumptions

**Assumption** **1.** *In the process of producing green products, two manufacturers need to increase research and development investment without increasing the basic costs of products. For simplicity of analysis, two manufacturers’ production costs are not considered here.*

**Assumption** **2.** *Both two manufacturers produce green products, and the greenness is*$e_{i}>0,i\in (1,2)$.

**Assumption** **3.** *When two manufacturers produce green products, the demand for green products by consumers is not only related to the prices of the manufacturers themselves, but also affected by the product greenness.*

**Assumption** **4.** *Referring to the research result of Ma et al. [12], it is assumed that the market demand for the products is a linear function of the retail prices and greenness of the products, which is*$d_{i}=\alpha -\beta p_{i}+\theta p_{j}-\frac{e_{i}{}^{2}}{2}+\tau e_{i},\;i\neq j,i\in (1,2),j\in (1,2)$.

**Assumption** **5.** *The research and development investment of green products has a law of diminishing marginal effect, which is represented by a quadratic function, that is, the research and development cost is*$\frac{e_{i}^{2}}{2}$. *The horizontal co-opetition relationship between the two manufacturers is a cooperative relationship. It is assumed that the two manufacturers conduct research and development separately, and independently bear their own research and development expenses.*

**Assumption** **6.** *The Stackelberg game is played among the three-stage supply chain, where two manufacturers are the leader, and one retailer is the follower.*

## 4. Model Construction and Analysis

### 4.1. Cooperative Supply Chain Decision Model Under Self-Interested

In the case of completely self-interested, two manufacturers and one retailer make product greenness and pricing decisions in the principle of maximizing their own profits. We study that the horizontal co-opetition relationship between the two manufacturers is a cooperative relationship, so two manufacturers will make decisions based on the maximization of the mutual profit of the two manufacturers when making greenness and pricing decisions. The profits of two manufacturers and one retailer are as follows:

(1) $$\Pi_{M}=w_{1}(\alpha -\beta p_{1}+\theta p_{2}+\tau e_{1})-\frac{e_{1}^{2}}{2}+w_{2}(\alpha -\beta p_{2}+\theta p_{1}+\tau e_{2})-\frac{e_{2}^{2}}{2}$$

(2) $$\Pi_{R}=(p_{1}-w_{1})(\alpha -\beta p_{1}+\theta p_{2}+\tau e_{1})+(p_{2}-w_{2})(\alpha -\beta p_{2}+\theta p_{1}+\tau e_{2})$$

**Proposition** **1.** *When two manufacturers and one retailer are completely self-interested, the unique optimal greenness,*$e_{i}{}^{NNN*}$, *wholesale prices,*$e_{i}{}^{NNN*}$, *and retail prices,*$p_{i}{}^{NNN*}$*are as follows:*$e_{i}{}^{NNN*}=\frac{\alpha \tau }{4\beta -4\theta -\tau^{2}}$, $w_{i}{}^{NNN*}=\frac{2\alpha }{4\beta -4\theta -\tau^{2}}$, $p_{i}{}^{NNN*}=\frac{3\alpha }{4\beta -4\theta -\tau^{2}}$.

**Proof.**  Please see Appendix A □

It is worth noting that Proposition 1 is conditional on *β*^2^ – *θ*^2^ > 0 and *τ*^2^ < 4(*β – θ*). Only when the above conditions are met, two manufacturers and one retailer have optimal pricing and greenness. At the same time, we find that the greenness, wholesale prices and retail prices of the products increase as the consumers’ sensitivity to the product greenness increases, so we can conclude that if the consumers are more sensitive to the product greenness, two manufacturers’ optimal greenness, wholesale prices and retail prices will increase. In this case, if two manufacturers and one retailer want to meet the consumers’ requirements for product greenness and obtain the expected profits, it is necessary to consider whether the improvement in greenness is initiated by two manufacturers, or is encouraged by the retailer’s encouragement, or the three parties agree to improve the product greenness, which is the three models discussed below.

From the above the proposition, the profits of two manufacturers and one retailer can be obtained as follows:

(3) $$\begin{array}{l} \Pi_{{}_{m1}}^{NNN*}=\Pi_{{}_{m2}}^{NNN*}=\frac{\alpha^{2}}{2(4\beta -4\theta -\tau^{2})},\\ \Pi_{{}_{M}}^{NNN*}=\frac{\alpha^{2}}{4\beta -4\theta -\tau^{2}},\\ \Pi_{{}_{R}}^{NNN*}=\frac{2\alpha^{2}(\beta -\theta )}{{\left(4\beta -4\theta -\tau^{2}\right)}^{2}},\\ \Pi_{sc}^{NNN*}=\frac{\alpha^{2}(6\beta -6\theta -\tau^{2})}{{\left(4\beta -4\theta -\tau^{2}\right)}^{2}} \end{array}$$

### 4.2. Cooperative Supply Chain Decision Model Under the Retailer with Altruistic Preference

When the retailer has an altruistic preference, it satisfies 0 < *ρ_r_* < 1. In order to help two manufacturers increase profits, the retailer no longer pursues the maximization of its own profit, but pursues the maximization of its utility. The retailer’s utility function is composed of the retailer’s own profit and manufacturers’ profits due of retailer’s altruistic preference:

(4) $$U_{R}=\Pi_{R}+\rho_{r}\Pi_{M}$$

Substituting the profit function Equation (1) of two manufacturers with no altruistic preferences into the retailer’s utility function Equation (4), we can obtain that:

(5) $$\begin{array}{l} U_{R}=(p_{1}-w_{1})(\alpha -\beta p_{1}+\theta p_{2}+\tau e_{1})+(p_{2}-w_{2})(\alpha -\beta p_{2}+\theta p_{1}+\tau e_{2})\\ +\rho_{r}\left[w_{1}(\alpha -\beta p_{1}+\theta p_{2}+\tau e_{1})-\frac{e_{1}^{2}}{2}+w_{2}(\alpha -\beta p_{2}+\theta p_{1}+\tau e_{2})-\frac{e_{2}^{2}}{2}\right] \end{array}$$

**Proposition** **2.** *When the retailer has altruistic preference, the unique optimal greenness,*$e_{i}{}^{NNR*}$*, wholesale prices,*$w_{i}{}^{NNR*}$*, and retail prices,*$p_{i}{}^{NNR*}$*, are as follows:*$e_{i}^{NNR*}=\frac{\alpha \tau }{4\beta -4\theta -\tau^{2}+4\rho_{r}(\theta -\beta )}$*,*$w_{i}^{NNR*}=\frac{2\alpha }{4\beta -4\theta -\tau^{2}+4\rho_{r}(\theta -\beta )}$*,*$p_{i}^{NNR*}=\frac{3\alpha (1-\rho_{r})}{4\beta -4\theta -\tau^{2}+4\rho_{r}(\theta -\beta )}$.

**Proof.**  Please see Appendix B □

It is worth noting that Proposition 2 is conditional on $\beta^{2}-\theta^{2}>0$ and $\tau^{2}<4(\beta -\theta )$. Only when the above conditions are met, two manufacturers and one retailer have optimal pricing and greenness. We can see from the optimal decision results of supply chain members that two manufacturers and one retailer need to consider the altruistic preference coefficients when making greenness and pricing decisions, and they make optimal decisions based on the retailer’s altruistic preference coefficient.

From the above the proposition, the profit of the retailer can be obtained, and the utilities of the manufacturers and the retailer are respectively as follows:

(6) $$\begin{array}{l} \Pi_{R}^{NNR*}=\frac{6(\beta -\theta )\alpha^{2}(\rho_{r}-1)(\rho_{r}-\frac{1}{3})}{{\left[4(\beta -\theta )(\rho_{r}-1)+\tau^{2}\right]}^{2}},\\ U_{M}^{NNR*}=-\frac{\alpha^{2}}{4(\beta -\theta )(\rho_{r}-1)+\tau^{2}},\\ U_{R}^{NNR*}=-\frac{\alpha^{2}2(\theta -\beta )(1+\rho_{r}{}^{2})+(-4\theta +4\beta +\tau^{2})\rho_{r}}{{\left[4(\beta -\theta )(\rho_{r}-1)+\tau^{2}\right]}^{2}}\\ U_{m1}^{NNR*}=U_{m2}^{NNA*}=-\frac{1}{2}\frac{\alpha^{2}}{4(\beta -\theta )(\rho_{r}-1)+\tau^{2}},\\ \Pi_{sc}^{NNR*}=\frac{\alpha^{2}\left[6(\beta -\theta ){\left(\rho_{r}+1\right)}^{2}-\tau^{2}\right]}{{\left[\tau^{2}+4(\beta -\theta )\rho_{r}+4(\theta -\beta )\right]}^{2}} \end{array}$$

**Proposition** **3.** *Only when the retailer’s altruistic preference coefficient satisfies*$0<\rho_{r}<\frac{1}{3}$*, the retailer will be profitable*.

**Proof.**  Please see Appendix C □

“The retailer will be profitable” means retail prices of retailer are higher than wholesale prices of the retailer. Proposition 3. indicates that:

(i) When the retailer has altruistic preference, its own profit is less than the profit when taking self-interested behavior. At this time, the retailer still adopts altruistic behavior, indicating that consumers have higher requirements for green products, hoping to buying products with higher levels of greenness, in order to encourage manufacturers to produce high greenness products and increase products sales. In the long term, the retailer is willing to sacrifice some of its own interest and cooperate with self-interested manufacturers to seek further development.
(ii) The profits of two manufacturers will increase with the increase of the retailer’s altruistic preference coefficient, and the retailer’s profit will continue to decrease with the increase of its own altruistic preference coefficient. For the sake of self-interested, the retailer will not increase the altruistic preference coefficient without limit.

**Proposition** **4.** *The wholesale prices, the product greenness, and the retail prices are positively correlated with the retailer’s altruistic preference coefficient.*

**Proof.**  Please see Appendix D □

Proposition 4 indicates that:

(i) In a cooperative supply chain, when the retailer has an altruistic preference, the retailer is willing to make efforts to improve the profits of the manufacturers.
(ii) The retailer, as the node that is in direct contact with consumers, can sensitively perceive changes in consumer demand. When the retailer perceive consumers in the market to pursue a higher product greenness, in order to encourage manufacturers to produce products with higher product greenness, and to establish an environmentally friendly image, the retailer is willing to get higher product greenness at higher wholesale prices, which can promote manufacturers to invest more in the development and production of green products. Improving the product greenness can increase the product sales and obtain more profits, and the increase of product sales will further promote the altruistic preferences of the retailer. When the retailer accepts higher wholesale prices, it will also increase retail prices for its own profit.
(iii) The retailer with altruistic behavior can encourage the manufacturers to produce products with higher level of greenness, establish a green company image, and improve the economic and ecological effects of the supply chain.

Therefore, two manufacturers will be more willing to cooperate with altruistic retailer. In the short term, although the retailer has a lower profit compared to self-interested behaviors due to altruistic preference, the retailer will still adopt altruistic preference in view of long-term economic effects and ecological effects.

### 4.3. Cooperative Supply Chain Decision Model Under Two Manufacturers with Altruistic Preferences

In the manufacturers-led supply chain structure, when two manufacturers conduct their own research and development of green products, the cooperative relationship can obtain higher wholesale prices than the competition. When two manufacturers consider altruistic preferences, Stackelberg game is played between the two manufacturers. Considering the cooperation between *M*_1_ and *M*_2_, the result of the game is: two manufacturers adopt altruistic preferences, and the altruistic preference coefficients are the same.

When two manufacturers have altruistic preferences, they satisfy 0 < *ρ_m_* < 1, and they pursue the maximization of the utility of both sides instead of mutual profit. Being the same as the previous definition of the utility function of the retailer, the mutual utility function of t two manufacturers is:

(7) $$U_{M}=\Pi_{M}+\rho_{m}\Pi_{R}$$

Substituting the respective profits functions of two manufacturers and the retailer for self-interest into Equation (7), we can obtain that:

(8) $$\begin{array}{l} U_{M}=w_{1}(\alpha -\beta P_{1}+\theta P_{2}+\tau e_{1})-\frac{e_{1}^{2}}{2}+w_{2}(\alpha -\beta P_{2}+\theta P_{1}+\tau e_{2})-\frac{e_{2}^{2}}{2}\\ +\rho_{m}\left[\left(P_{1}-w_{1})(\alpha -\beta P_{1}+\theta P_{2}+\tau e_{1})+(P_{2}-w_{2})(\alpha -\beta P_{2}+\theta P_{1}+\tau e_{2}\right)\right] \end{array}$$

**Proposition** **5.** *when two manufacturers have altruistic preferences, the unique optimal greenness,*$e_{i}{}^{MMN*}$*, wholesale prices,*$w_{i}{}^{MMN*}$*, and retail prices,*$p_{i}{}^{MMN*}$*, are as follows:*$e_{i}^{MMN*}=\frac{\alpha \tau }{4\beta -4\theta -\tau^{2}-2\rho_{m}(\beta -\theta )}$*,*$w_{i}^{MMN*}=\frac{2\alpha (1-\rho_{m})}{4\beta -4\theta -\tau^{2}-2\rho_{m}(\beta -\theta )}$*,*$p_{i}^{MMN*}=\frac{\alpha (3-2\rho_{m})}{4\beta -4\theta -\tau^{2}-2\rho_{m}(\beta -\theta )}$.

**Proof.**  Please see Appendix E □

It is worth noting that Proposition 5 is conditional on $\beta^{2}-\theta^{2}>0$, $(\rho_{m}{}^{2}-2\rho_{m}+\frac{1}{2})\beta \tau^{2}+(2-\rho_{m})(\beta^{2}-\theta^{2})<0$ and $\tau^{4}\left[2\beta^{2}\rho_{m}(\rho_{m}-2)+\beta^{2}-\theta^{2}\right]+\tau^{2}\left[4\beta^{2}(1-\rho_{m})+\rho_{m}{}^{2}(\beta^{2}-\theta^{2})+4\theta (\beta -\theta )\right]-4{\left(\rho_{m}-2\right)}^{2}{\left(\beta^{2}-\theta^{2}\right)}^{2}>0$. Only when the above conditions are met, two manufacturers and one retailer have optimal pricing and greenness. We can see from the optimal decision results that two manufacturers and one retailer need to put altruistic preference coefficients of the manufacturers into consideration when pricing and making greenness decisions.

The profits and the utility values of two manufacturers and the utility value of the retailer can be obtained by above the proposition:

(9) $$\begin{array}{l} \Pi_{M}^{MMN*}=\frac{\alpha^{2}(4\beta -4\theta -\tau^{2}-4\rho_{m}(\beta -\theta )}{{\left[4\beta -4\theta -\tau^{2}-2\rho_{m}(\beta -\theta )\right]}^{2}},\\ \Pi_{m1}^{MMN*}=\Pi_{m2}^{MMN*}=\frac{1}{2}\frac{\alpha^{2}(4\beta -4\theta -\tau^{2}-4\rho_{m}(\beta -\theta ))}{{\left[4\beta -4\theta -\tau^{2}-2\rho_{m}(\beta -\theta )\right]}^{2}}\\ U_{M}^{MMN*}=\frac{\alpha^{2}}{4\beta -4\theta -\tau^{2}-4\rho_{m}(\beta -\theta )},\\ U_{R}^{MMN*}=\frac{2\alpha^{2}(\beta -\theta )}{{\left[4\beta -4\theta -\tau^{2}-2\rho_{m}(\beta -\theta )\right]}^{2}},\\ \Pi_{sc}^{MMN*}=\frac{\alpha^{2}\left[2(\beta -\theta )(3-2\rho_{m})-\tau^{2}\right]}{{\left[\tau^{2}+4(\theta -\beta )+2\rho_{m}(\beta -\theta )\right]}^{2}} \end{array}$$

**Proposition** **6.** *In cooperative supply chain, if two manufacturers have altruistic preferences, the product greenness is positively correlated with the altruistic preference coefficients of two manufacturers, while the retail prices and the wholesale prices are negatively correlated with the altruistic preference coefficients of two manufacturers.*

**Proof.**  Please see Appendix F □

Proposition 6 shows that when two manufacturers have altruistic preferences, in order to improve company image and increase product sales, two manufacturers will actively increase the product greenness, while considering the interest of the retailer, and they will sell to the retailer at lower wholesale prices. And if the product sales increase, altruistic preferences of the manufacturers will be encouraged. Two manufacturers are selling products to the retailer at lower prices while increasing product greenness. Although they reduce the profit of the manufacturers, they increase the utility of the manufacturers.

**Proposition** **7.** *The relationship between the product greenness when two manufacturers have altruistic preferences and when the retailer has an altruistic preference is related to the altruistic preference coefficients they have, and the product greenness in both cases is higher than there are no altruistic preferences.*

**Proof.**  Please see Appendix G □

Proposition 7 indicates that:

(i) When *ρ_m_* = 2*ρ_r_*, two manufacturers have altruistic preferences and the retailer has an altruistic preference that encourage two manufacturers to produce higher product greenness, and the product greenness in these two altruistic preference models is the same.
(ii) When *ρ_m_* > 2*ρ_r_*, compared with the retailer who has an altruistic preference, two manufacturers have altruistic preferences that encourage the manufacturers to produce higher product greenness and sell them to the retailer at lower wholesale prices, which is more suitable for the promotion period of green products.
(iii) When *ρ_m_* < 2*ρ_r_*, compared with two manufacturers who have altruistic preferences, the retailer with altruistic preference encourages two manufacturers to produce higher product greenness, which is more suitable for consumers to be more sensitive to the product greenness and the competition of the retailers is fierce.
(iv) Regardless of whether two manufacturers or the retailer has altruistic preferences, in order to improve economic and social benefits, the manufacturers will increase the green level of products to attract consumers and increase the product sales.

**Proposition** **8.** *when two manufacturers have altruistic preferences, the altruistic preference coefficients will satisfy*$1-\sqrt{\frac{1}{2}}<\rho_{m}<1$.

**Proof.**  Please see Appendix H □

Proposition 8 indicates that when two manufacturers have altruistic preferences, if two manufacturers have the lower altruistic preference coefficients, the manufacturers will increase the wholesale prices of the products in order to maintain their own interests after improving the product greenness. Although the retailer receives higher levels of green products from manufacturers, wholesale prices will be higher. When products are sold in the market, higher retail prices will cause consumers to regress, which results in lower products sales and lower retailer’ profit. If two manufacturers do not increase the altruistic preference coefficients, the retailer will re-find the partner for its interest. Therefore, if two manufacturers want to adopt altruistic preferences in the supply chain, the higher level of altruistic preference coefficients will be adopted for the consideration of the retailer profit.

### 4.4. Cooperative Supply Chain Decision Model Under Two Manufacturers and One Retailer All with Altruistic Preferences

When two manufacturers and one retailer all have altruistic preferences, they satisfy $0<\rho_{m}<1,0<\rho_{r}<1$. When making greenness and pricing decisions, considering the interest of the retailer, two manufacturers are no longer making pricing and greening decisions based on the principle of maximizing the interests of both, but based on their mutual utility. Similarly, the retailer will also price at its utility maximization. Referring to the definition of the utility function of the previous retailer, the utility functions of two manufacturers and one retailer are as follows:

(10) $$U_{M}=\Pi_{M}+\rho_{m}\Pi_{R}$$

(11) $$U_{R}=\Pi_{R}+\rho_{R}\Pi_{M}$$

Substituting Equations (1) and (2) into Equations (10) and (11), respectively, we can obtain that:

(12) $$\begin{array}{l} U_{M}=w_{1}(\alpha -\beta P_{1}+\theta P_{2}+\tau e_{1})-\frac{e_{1}^{2}}{2}+w_{2}(\alpha -\beta P_{2}+\theta P_{1}+\tau e_{2})-\frac{e_{2}^{2}}{2}\\ +\rho_{m}\left[\left(P_{1}-w_{1})(\alpha -\beta P_{1}+\theta P_{2}+\tau e_{1})+(P_{2}-w_{2})(\alpha -\beta P_{2}+\theta P_{1}+\tau e_{2}\right)\right] \end{array}$$

(13) $$\begin{array}{l} U_{R}=(P_{1}-w_{1})(\alpha -\beta P_{1}+\theta P_{2}+\tau e_{1})+(P_{2}-w_{2})(\alpha -\beta P_{2}+\theta P_{1}+\tau e_{2})\\ +\rho_{R}\left[w_{1}(\alpha -\beta P_{1}+\theta P_{2}+\tau e_{1})-\frac{e_{1}^{2}}{2}+w_{2}(\alpha -\beta P_{2}+\theta P_{1}+\tau e_{2})-\frac{e_{2}^{2}}{2}\right] \end{array}$$

**Proposition** **9.** *When two manufacturers and one retailer have altruistic preferences, the unique optimal greenness,*$e_{i}{}^{MMR*}$*, wholesale prices,*$w_{i}{}^{MMR*}$*, and retail prices,*$p_{i}{}^{MMR*}$*, are as follows:*$e_{i}^{MMR*}=-\frac{{\left(\rho_{m}\rho_{r}-1\right)}^{2}\tau \alpha }{{\left(\rho_{m}\rho_{r}-1\right)}^{2}\tau^{2}-2(\beta -\theta )\left[\rho_{m}(\rho_{r}{}^{2}-1)+2(1-\rho_{r})\right]}$*,*$w_{i}^{MMR*}=-\frac{2(\rho_{m}-1)\alpha }{{\left(\rho_{m}\rho_{r}-1\right)}^{2}\tau^{2}-2(\beta -\theta )\left[\rho_{m}(\rho_{r}{}^{2}-1)+2(1-\rho_{r})\right]}$*,*$p_{i}^{MMR*}=-\frac{(\rho_{r}-1)\left[-3+(\rho_{r}+2)\rho_{m}\right]\alpha }{{\left(\rho_{m}\rho_{r}-1\right)}^{2}\tau^{2}-2(\beta -\theta )\left[\rho_{m}(\rho_{r}{}^{2}-1)+2(1-\rho_{r})\right]}$.

**Proof.**  Please see Appendix I □

It is worth noting that Proposition 9 is conditional on $\beta^{2}-\theta^{2}>0$ and ${\left(\rho_{m}\rho_{r}-1\right)}^{2}\tau^{2}+2(\beta +\theta )\left[\rho_{m}(1+\rho_{r})-2\right](1-\rho_{r})<0$. Only when the above conditions are met, two manufacturers and one retailer have optimal pricing and greenness. We can see from the results of optimal decisions that when making decisions of prices and greenness, two manufacturers and the retailer should take the altruistic preference coefficients into consideration.

The profits and the utility value of the manufacturers and the profit and the utility value of the retailer can be obtained by above the proposition:

(14) $$\begin{array}{l} \Pi_{m1}^{MMR*}=\Pi_{m2}^{MMR*}=\frac{1}{2}\Pi_{M}^{MMR*},\\ \Pi_{M}^{MMR*}=\frac{(1-\rho_{m}\rho_{r})\alpha^{2}\left\{\rho_{m}\rho_{r}\tau^{2}(\rho_{m}\rho_{r}-1)(\rho_{m}\rho_{r}-2)+4(\beta -\theta )\left[\rho_{m}(\rho_{r}-1)+\rho_{r}+1\right]\right\}}{{\left\{{\left(\rho_{m}\rho_{r}-1\right)}^{2}\tau^{2}-2(\beta -\theta )\left[\rho_{m}(\rho_{r}{}^{2}-1)+2(1-\rho_{r})\right]\right\}}^{2}}\\ \Pi_{R}^{MMR*}=\frac{2\alpha^{2}(1-\rho_{r})(\beta -\theta )(\rho_{m}\rho_{r}-1)(\rho_{m}\rho_{r}{}^{2}+(\rho_{m}-3)\rho_{r}+1)}{{\left\{{\left(\rho_{m}\rho_{r}-1\right)}^{2}\tau^{2}-2(\beta -\theta )\left[\rho_{m}(\rho_{r}{}^{2}-1)+2(1-\rho_{r})\right]\right\}}^{2}},\\ U_{M}^{MMR*}=\frac{\alpha^{2}{\left(\rho_{m}\rho_{r}-1\right)}^{2}}{2(\beta -\theta )\left[\rho_{m}(\rho_{r}{}^{2}-1)+2(1-\rho_{r})\right]-{\left(\rho_{m}\rho_{r}-1\right)}^{2}\tau^{2}}\\ U_{R}^{MMR*}=\frac{\alpha^{2}{\left(\rho_{m}\rho_{r}-1\right)}^{2}\{[2(\beta -\theta )+2\tau^{2}\rho_{m}]\rho_{r}{}^{2}+2(\beta -\theta )-\tau^{2}\rho_{m}{}^{2}\rho_{r}{}^{3}\}}{{\left\{{\left(\rho_{m}\rho_{r}-1\right)}^{2}\tau^{2}-2(\beta -\theta )\left[\rho_{m}(\rho_{r}{}^{2}-1)+2(1-\rho_{r})\right]\right\}}^{2}},\\ \Pi_{sc}^{MMR*}=\frac{\alpha^{2}[2(\beta -\theta ){\left(\rho_{r}-1\right)}^{2}(\rho_{m}\rho_{r}+2\rho_{m}-3)-{\left(\rho_{m}\rho_{r}-1\right)}^{3}\tau^{2}]}{{\left\{{\left(\rho_{m}\rho_{r}-1\right)}^{2}\tau^{2}-2(\beta -\theta )\left[\rho_{m}(\rho_{r}{}^{2}-1)+2(1-\rho_{r})\right]\right\}}^{2}} \end{array}$$

**Proposition** **10.** *When two manufacturers and one retailer all have altruistic preferences, the product greenness is positively correlated with the retailer’s altruistic preference coefficient, and retail prices and wholesale prices are negatively correlated with altruistic preference coefficients of two manufacturers.*

**Proof.**  Please see Appendix J □

Proposition 10 indicates that the retailer’s preference coefficient has a significant effect on promoting the product greenness, while two manufacturers’ altruistic preference coefficients have a significant effect on promoting two manufacturers to lower wholesale prices and the retailer to lower retail prices.

**Proposition** **11.** *Compared with the two manufacturers have altruistic preferences and the retailer has an altruistic preference, when two manufacturers and one retailer all have altruistic preferences, the wholesale prices of the products are related to the altruistic preference coefficients of two manufacturers.*

**Proof.**  Please see Appendix K □

Proposition 11 indicates that:

(i) When $\rho_{m}=\frac{2(\beta -\theta )}{\tau^{2}}$, the wholesale prices of two manufacturers with altruistic preferences are equal to the wholesale prices of two manufacturers and the retailer all with altruistic preferences.
(ii) When $\rho_{m}<\frac{2(\beta -\theta )}{\tau^{2}}$, the wholesale prices are lower when two manufacturers and the retailer all have altruistic preferences, compared with two manufacturers with altruistic preferences.
(iii) When $\rho_{m}>\frac{2(\beta -\theta )}{\tau^{2}}$, the wholesale prices are lower when two manufacturers have altruistic preferences, compared with two manufacturers and the retailer all with altruistic preferences.

**Proposition** **12.** *The product greenness is higher when two manufacturers and one retailer all have altruistic preferences, compared with two manufacturers with altruistic preferences.*

**Proof.**  Please see Appendix L □

Proposition 12 indicates that higher product greenness will be produced by manufacturers in the situation that two manufacturers and the retailer have altruistic preference than two manufacturers have altruistic preferences.

**Proposition** **13.** *Interestingly, we find that when two manufacturers and one retailer all have altruistic preferences, product greenness, wholesale prices, and retailer prices change no longer appear a certain relationship with the altruistic preference coefficients of two manufacturers or the retailer’s altruistic preference coefficient. Rather, when two manufacturers and the retailer adopt different altruistic preference coefficients, the relationship between relevant decision variables and the altruistic preference coefficients of two manufacturers and one retailer is also changing.*

**Proof.**  From the above the conclusion, we can obtain that: □

**Situation** **1.** *There is an uncertainty relationship between the product greenness when two manufacturers and one retailer all have altruistic preferences and the product greenness when the retailer has an altruistic preference.*

**Proof.**  Please see Appendix M □

Situation 1 indicates that:

(i) When $\rho_{m}\rho_{r}{}^{2}-\frac{3}{2}\rho_{r}+\frac{1}{2}=0,e_{i}^{MMR*}=e_{i}^{NNR*}>e_{i}^{NNN*}$.
(ii) When $\rho_{m}\rho_{r}{}^{2}-\frac{3}{2}\rho_{r}+\frac{1}{2}<0,e_{i}^{MMR*}>e_{i}^{NNR*}>e_{i}^{NNN*}$.
(iii) When $\rho_{m}\rho_{r}{}^{2}-\frac{3}{2}\rho_{r}+\frac{1}{2}>0,e_{i}^{MMR*}>e_{i}^{NNR*}>e_{i}^{NNN*}$.

**Situation** **2.** *The relation between product greenness and altruistic preference coefficient of the retailer is uncertain, when two manufacturers and one retailer all have altruistic preferences.*

**Proof.**  Please see Appendix N □

Situation 2 indicates that:

(i) When $\rho_{m}\rho_{r}{}^{2}+(\rho_{m}-3)\rho_{r}+1=0$, the product greenness is irrelevant with two manufacturers’ altruistic preference coefficients.
(ii) When $\rho_{m}\rho_{r}{}^{2}+(\rho_{m}-3)\rho_{r}+1>0$, the product greenness is positively correlated with two manufacturers’ altruistic preference coefficients.
(iii) When $\rho_{m}\rho_{r}{}^{2}+(\rho_{m}-3)\rho_{r}+1<0$, the product greenness is negatively correlated with two manufacturers’ altruistic preference coefficients.

**Situation** **3.** *The relation between the wholesale prices of products and altruistic preference coefficient of the retailer is uncertain, when two manufacturers and one retailer all have altruistic preferences.*

**Proof.**  Please see Appendix O □

Situation 3 indicates that:

(i) When $\rho_{m}=\frac{2(\beta -\theta )}{\tau^{2}}$, the wholesale prices are irrelevant with altruistic preference coefficient of the retailer.
(ii) When $\rho_{m}>\frac{2(\beta -\theta )}{\tau^{2}}$, the wholesale prices are negatively correlated with altruistic preference coefficient of the retailer.
(iii) When $\rho_{m}<\frac{2(\beta -\theta )}{\tau^{2}}$, the wholesale prices are positively correlated with altruistic preference coefficient of the retailer.

**Situation** **4.** *The relation between the retail prices of products and altruistic preference coefficient of the retailer is uncertain, when two manufacturers and one retailer all have altruistic preferences.*

**Proof.**  Please see Appendix P □

Situation 4 indicates that:

(i) When $(\rho_{m}\rho_{r}-1)(\rho_{m}\rho_{r}+3)+4(1-\rho_{m}\rho_{r})\rho_{m}-2\rho_{m}(\beta -\theta ){\left(\rho_{r}-1\right)}^{2}=0$, the retail prices are irrelevant with altruistic preference coefficient of the retailer.
(ii) When $(\rho_{m}\rho_{r}-1)(\rho_{m}\rho_{r}+3)+4(1-\rho_{m}\rho_{r})\rho_{m}-2\rho_{m}(\beta -\theta ){\left(\rho_{r}-1\right)}^{2}>0$, the retail prices are negatively correlated with altruistic preference coefficient of the retailer.
(iii) When $(\rho_{m}\rho_{r}-1)(\rho_{m}\rho_{r}+3)+4(1-\rho_{m}\rho_{r})\rho_{m}-2\rho_{m}(\beta -\theta ){\left(\rho_{r}-1\right)}^{2}<0$, the retail prices are positively correlated with altruistic preference coefficient of the retailer.

Proposition 13 indicates that:

(i) When two manufacturers and one retailer all have altruistic preferences and adopt different preference coefficients respectively, the product greenness is not always higher than that of the situation that only the retailer has altruistic preference, but certainly higher than that of the situation that two manufacturers have altruistic preferences.
(ii) When two manufacturers and one retailer all have different altruistic preference coefficients, the relationship between product greenness, wholesale prices, and retail prices and altruistic preference coefficients appears to be inconsistent with the situations when the manufacturers have altruistic preferences and when the retailer has an altruistic preference.

## 5. Numerical Analysis

In order to verify the correctness and efficiency of the models, the impact of the parameter variables on the decision and profits of supply chain members is verified by numerical simulation. According to the above model analysis, It is worth noting that the four models are conditional on β>θ and $\tau^{2}<4(\beta -\theta )$, therefore, it is assumed that the potential market demand quantity is *α* = 50, the price elasticity of demand is *β* = 0.6, the cross-price sensitivity coefficient is *θ* = 0.3, the green sensitivity coefficient of consumers is *τ* = 0.2.

### 5.1. The Impact of Altruistic Preference of the Retailer on Pricing, Greenness and Profits

#### 5.1.1. The Impact of Altruistic Preference on Greenness and Pricing Decisions

**Figure 2:**
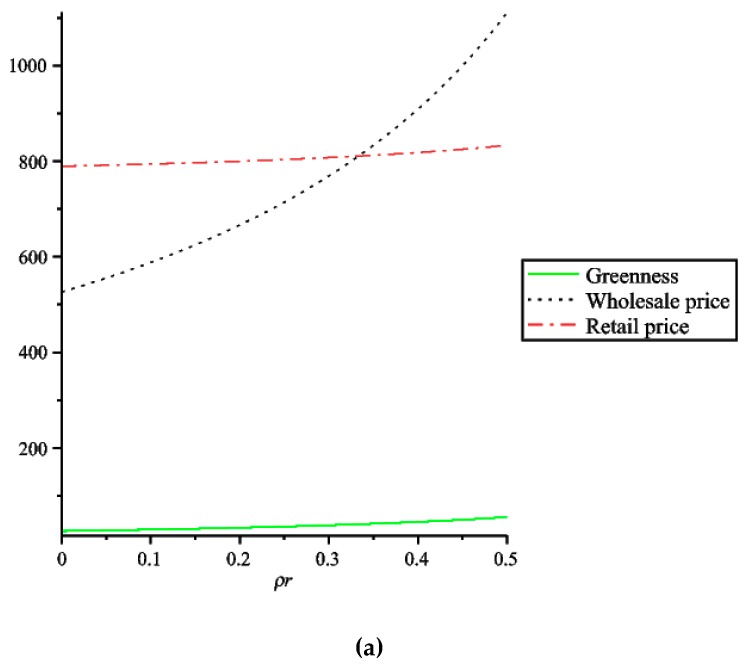

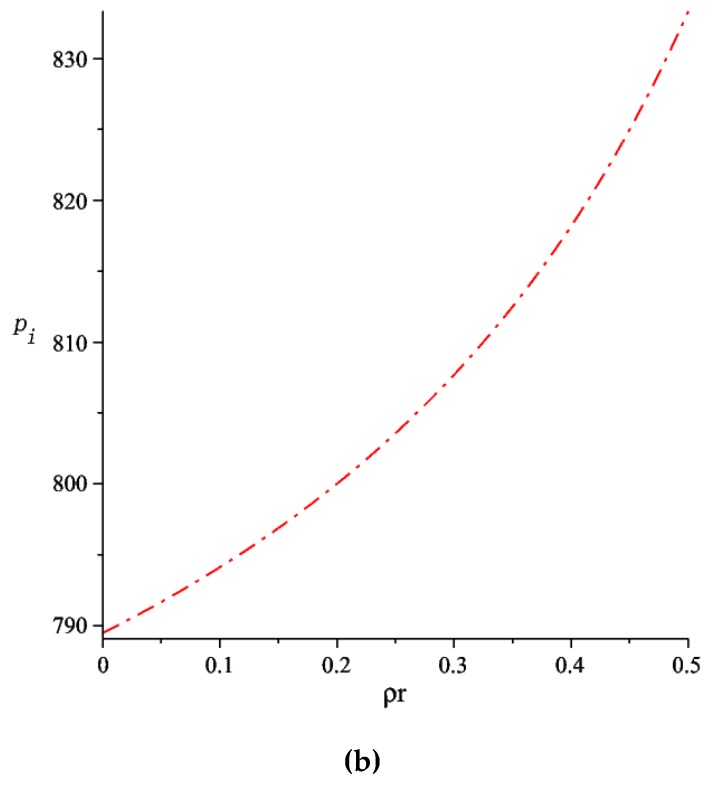
The effect of retailer’s altruistic preference coefficient on the decision of supply chain members. (**a**) The impact of retailer’s altruistic preference coefficient on greenness, wholesale prices and retail prices. (**b**) The impact of the retailer’s altruistic preference coefficient on retail prices.

It can be concluded from Figure 2 that when the retailer has an altruistic preference, if the retailer increases the altruistic preference coefficient, the product greenness and the wholesale prices will increase. Due to the increase of wholesale prices, the retailer will increase the retail prices when it sells products for its own benefit. 

From Figure 2a we can see that in the case of the retailer with altruistic preference, when the retailer’s altruistic preference coefficient increases, although the retailer’s retail prices will increase, the increase in retail prices is much smaller than the increase in wholesale prices, which means that when the retailer has an altruistic preference, the retailer will encourage two manufacturers to produce greener products for the profits of two manufacturers, the retailer is willing to buy products at higher prices, but the increase of prices is smaller when selling to consumers. This approach can attract consumers, increase product sales, and increase ecological benefits. From Figure 2 we can also see that when the retailer’s altruistic preference coefficient satisfies $\rho_{r}=\frac{1}{3}$, the retail prices are equal to the wholesale prices. If the retailer increases the altruistic preference coefficient, its own profit will be harmed. For the sake of self-interest, the retailer’s altruistic preference coefficient will satisfy $\rho_{r}<\frac{1}{3}$.

#### 5.1.2. The Impact of Altruistic Preference on Supply Chain Profits

**Figure 3:**
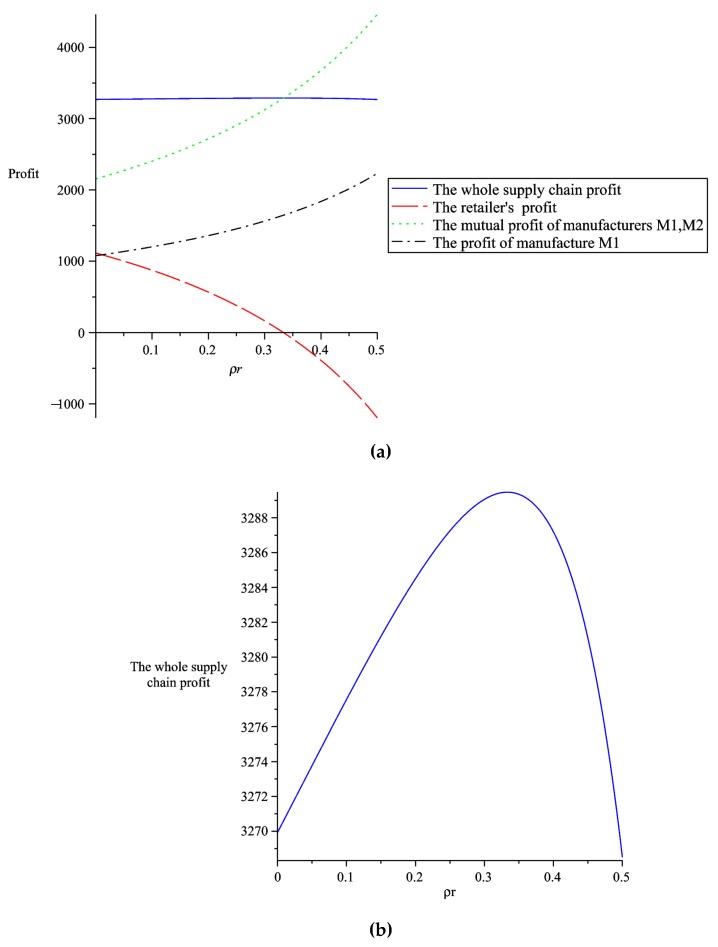
The effect of the retailer’s altruistic preference coefficient on supply chain profits. (**a**) The impact of the retailer’s altruistic preference coefficient on supply chain profits. (**b**) The impact of the retailer’s altruistic preference coefficient on the whole supply chain profit.

From Figure 3b, we can see that when the retailer’s altruistic preference coefficient satisfies $\rho_{r}<\frac{1}{3}$, the whole supply chain profit increases with the increase of the retailer’s altruistic preference coefficient. However, when $\rho_{r}>\frac{1}{3}$, the whole supply chain profit decreases with the increase of the retailer’s altruistic preference coefficient, indicating that only the retailer’s altruistic preference coefficient satisfies $\rho_{r}<\frac{1}{3}$, which is beneficial to the retailer’s own interest. From Figure 3a, we can see that as the retailer’s altruistic preference coefficient increases, the retailer’s own profit is declining, while two manufacturers’ mutual profit and their respective profits are constantly increasing, which indicates that the retailer with altruistic preference is willing to sacrifice its own profit for the profits of two manufacturers. The retailer’s altruistic behavior encourages two manufacturers to increase the greenness of their products. Due to two manufacturers need to invest more in improving the product greenness, two manufacturers will increase their wholesale prices in consideration of their own interests. At the same time, due to the retailer’s altruistic preference, the retail prices will only increase slightly, which will increase the demand of the products, so that the profits and the social effect of two manufacturers are increased.

### 5.2. The Impact of Altruism Preference Coefficient of Two Manufacturers on Pricing, Greenness and Profits

#### 5.2.1. The Impact of Altruistic Preference on Greenness and Pricing Decisions

**Figure 4:**
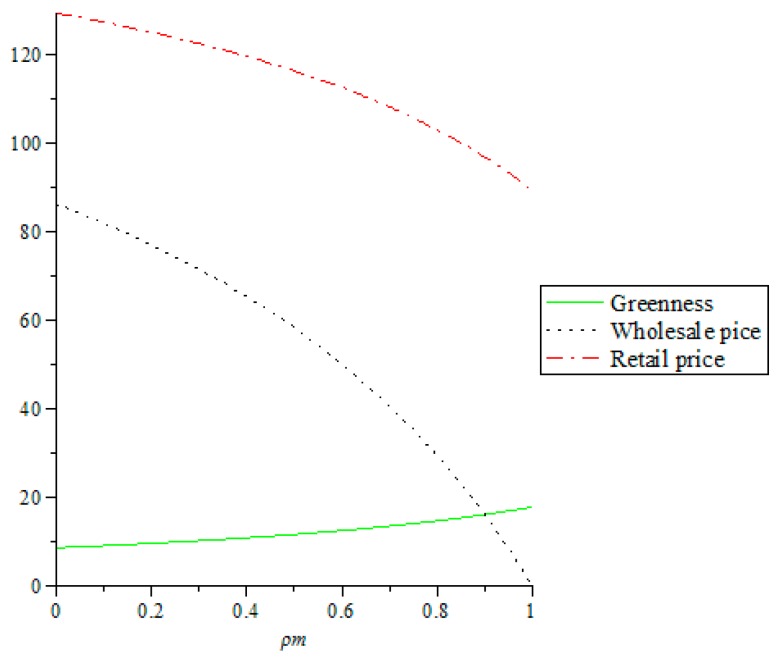
The impact of altruistic preference coefficients of two manufacturers on product greenness, wholesale prices and retail prices.

It can be seen from Figure 4. that when two manufacturers have altruistic preferences, the product greenness increases as the manufacturers’ altruistic preference coefficients increase, while the wholesale prices and the retail prices are decreasing as the manufacturers’ altruistic preference coefficients increase, which explains when two manufacturers have altruism preferences, taking into account the interest of the retailer, two manufacturers will actively improve the product greenness, while setting lower wholesale prices. When the prices of the products obtained by the retailer are low, after the Stackelberg game, the retail prices will be lower, which will attract more consumers and increase the product sales, thereby increasing the profit of the retailer. Considering that consumers have green preferences, the manufacturers will increase the greenness, which will increase the sales of their products, and increase their profits and social benefits.

#### 5.2.2. The impact of altruistic preference on supply chain profits

**Figure 5:**
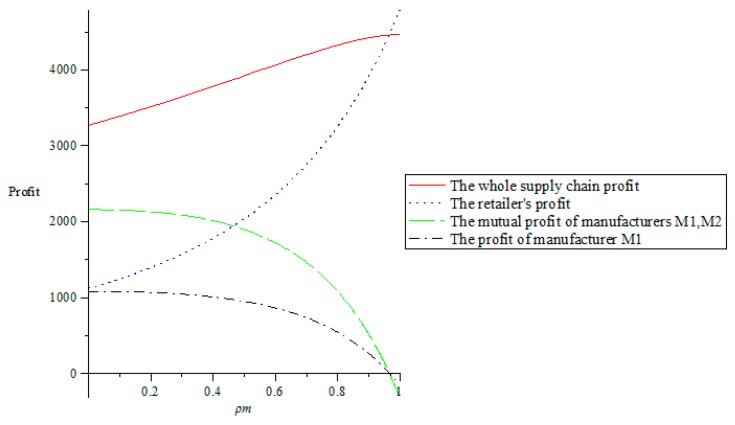
The impact of altruistic preference coefficients of two manufacturers on supply chain profits.

As can be seen from Figure 5, when two manufacturers have altruistic preferences, the whole supply chain profit and the retailer’s profit increase as the manufacturers’ altruistic preference coefficients increase, while the profits of two manufacturers and the respective profit of two manufacturers decrease as the manufacturers’ altruistic preference coefficients increase, indicating that when two manufacturers have altruistic preferences, two manufacturers will consider the interest of the retailer. Through the above analysis, when two manufacturers have altruistic preferences, the three parties are beneficial, however, when two manufacturers’ altruistic preference coefficients satisfy $\rho_{m}\in [0,0.5)$, the mutual profit of two manufacturers is greater than the retailer’s profit, and when two manufacturers’ altruistic preference coefficients satisfy $\rho_{m}\in \left[0.5,1\right]$, the retailer’s benefit is greater than the mutual profit of two manufacturers, and two manufacturers’ respective profit is always less than the retailer’s profit.

### 5.3. The Impact of Altruistic Preference Coefficients of Two Manufacturers and One Retailer on Pricing, Greenness and Profits

#### 5.3.1. The Impact of Altruistic Preference on Greenness and Pricing Decisions

**Figure 6:**
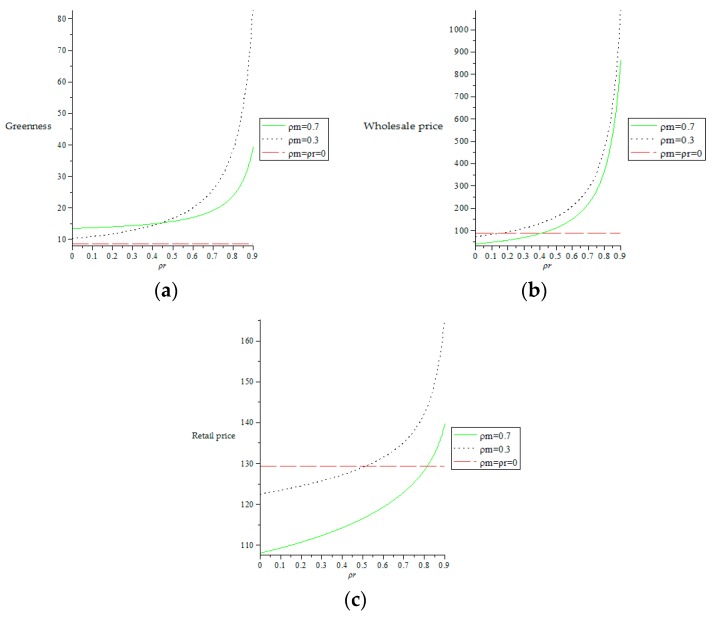
The effect of altruistic preference coefficients of two manufacturers and the retailer on the decisions of supply chain members. (**a**) The impact of altruistic preference coefficients of two manufacturers and the retailer on greenness. (**b**) The impact of altruistic preference coefficients of two manufacturers and the retailer on wholesale prices. (**c**) The impact of altruistic preference coefficients of two manufacturers and the retailer on retail prices.

As shown in Figure 6a, the straight dotted line represents the product greenness when two manufacturers and one retailer have no altruistic preferences. As can be seen from Figure 6a, when two manufacturers and one retailer all have altruistic preferences, the product greenness is higher than there are no altruistic preferences. When two manufacturers’ altruistic preference coefficients are certain, the product greenness increases as the retailer’s altruistic preference coefficient increases, but when the retailer’s altruistic preference coefficient is less than 0.44, higher manufacturers’ altruistic preference coefficients can lead to higher greenness. When the retailer’s altruistic preference coefficient is greater than 0.44, higher retailer altruism preference coefficient can lead to higher products greenness. This indicates that two manufacturers’ altruistic preference coefficients have a greater impact on the product greenness.

As shown in Figure 6b,c, the straight dotted line represents the wholesale prices and retail prices when two manufacturers and one retailer have no altruistic preferences. It can be seen from Figure 6b,c that when the altruistic preference coefficients of two manufacturers and one retailer are within a certain range, the wholesale prices and the retail prices are lower than there are no altruistic preferences. When two manufacturers’ altruistic preference coefficients are certain, the wholesale prices and retail prices increase as the retailer’s altruistic preference coefficient increases, which shows that when the retailer’s altruistic preference coefficient increases, the retailer will consider the interests of two manufacturers. At this time, the retailer is willing to sacrifice some of its own interest and obtain the products at higher wholesale prices. Considering the interest, the retailer will sell products at higher retail prices. When the retailer’s altruistic preference coefficient is certain, the wholesale prices and retail prices decrease as two manufacturers’ altruistic preference coefficients increase, which shows that when two manufacturers’ altruistic preference coefficients increase, two manufacturers will consider the interest of the retailer and will be willing to sacrifice some of their own interests and sell them to the retailer at lower wholesale prices. At the same time, the retailer’s Stacklberg game will also sell the products at lower retail prices to increase the products sales, which in turn promotes the altruistic preferences of the manufacturers.

#### 5.3.2. The Impact of Altruistic Preference on Supply Chain Profits

**Figure 7:**
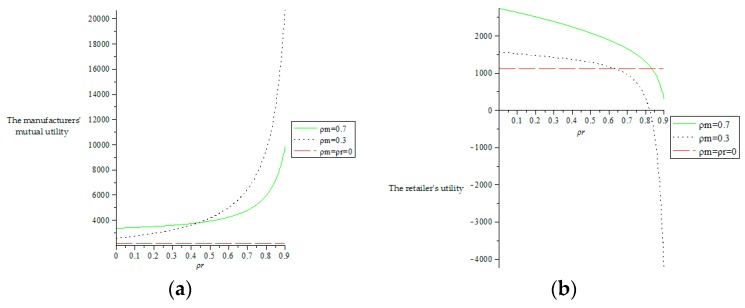

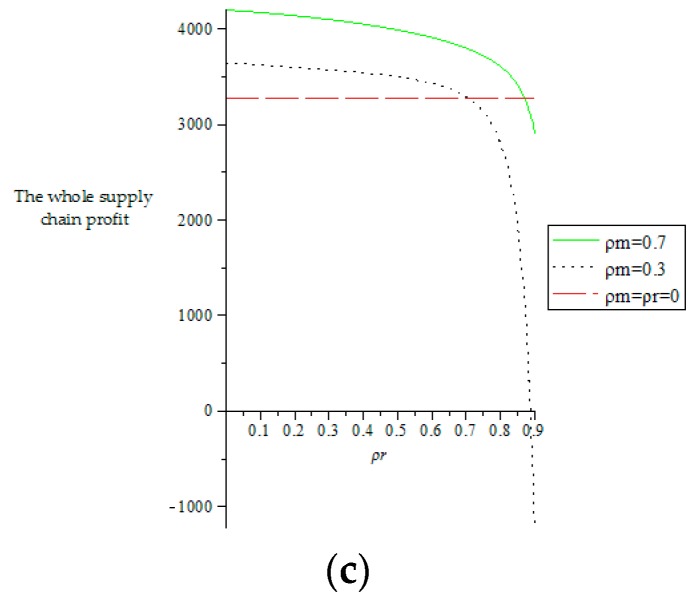
The effect of altruistic preference coefficients of two manufacturers and the retailer on the utilities and profits of the supply chain members. (**a**) The impact of altruistic preference coefficients of two manufacturers and the retailer on the mutual utility of two manufacturers. (**b**) The impact of altruistic preference coefficients of two manufacturers and the retailer on the utility of the retailer. (**c**) The impact of altruistic preference coefficients of two manufacturers and the retailer on the whole supply chain profit.

As shown in Figure 7a, the straight dotted line represents the mutual utility of two manufacturers when two manufacturers and one retailer have no altruistic preferences. As can be seen from Figure 7a, when two manufacturers and one retailer all have altruistic preferences, the mutual utility of two manufacturers is greater than the case of no altruistic preferences. When two manufacturers’ altruistic preference coefficients are certain, the mutual utility of two manufacturers increases as the retailer’s altruistic preference coefficient increases, but when the retailer’s altruistic preference is less than 0.44, higher manufacturers’ altruistic preferences can lead to higher manufacturers’ mutual utility, when the retailer’s altruism preference coefficient is greater than 0.44, the higher manufacturers’ altruistic preferences can bring lower manufacturers’ mutual utility, which means that the manufacturers’ altruistic preferences have greater effect on the mutual utility of two manufacturers.

As shown in Figure 7b, the straight dotted line represents the utility of the retailer when two manufacturers and one retailer have no altruistic preferences. As can be seen from Figure 7b, when the retailer’s altruistic coefficient is certain, the retailer’s utility decreases as the retailer’s altruistic preference coefficient increases, which is because when the retailer increases the altruistic preference coefficient, the retailer gives more profit to two manufacturers. When two manufacturers’ altruistic coefficients are certain, the retailer’s utility increases as the manufacturers’ altruistic preference coefficients increase, which indicates that when two manufacturers increase the altruistic preference coefficients, two manufacturers give more profit to the retailer. At the same time, we can also observe from the Figure 7b that when two manufacturers’ altruistic preference coefficients are low, the retailer’s utility appears to be less than the retailer’s utility when there are no altruistic preferences, and even a negative value appears, which indicates that the altruistic preference coefficients of two manufacturers and one retailer are within a certain range, so that the utility of the retailer is greater than the retailer’s utility when there are no altruistic preferences. As can be seen by comparing Figure 7a,b, the manufacturers’ altruistic preferences have a greater effect on the utilities of two manufacturers and the retailer.

From the Figure 7c, we can see that the relationship between the whole supply chain profit and the altruistic coefficients of two manufacturers and the retailer is the same as the relationship between the retailer’s utility and the altruistic coefficients of two manufacturers and the retailer, so we can conclude that two manufacturers’ altruistic preference coefficients have a greater effect on the whole supply chain profit.

From the above analysis, we can see that when the altruistic preference coefficients of two manufacturers and the retailer are continuously changing in the interval [0,0.9]. In order to facilitate the analysis, we analyze the change in the relationship between the variables and the altruistic preference coefficients when the altruistic preference coefficient is in the interval [0,0.5], without affecting the final result.

**Figure 8:**
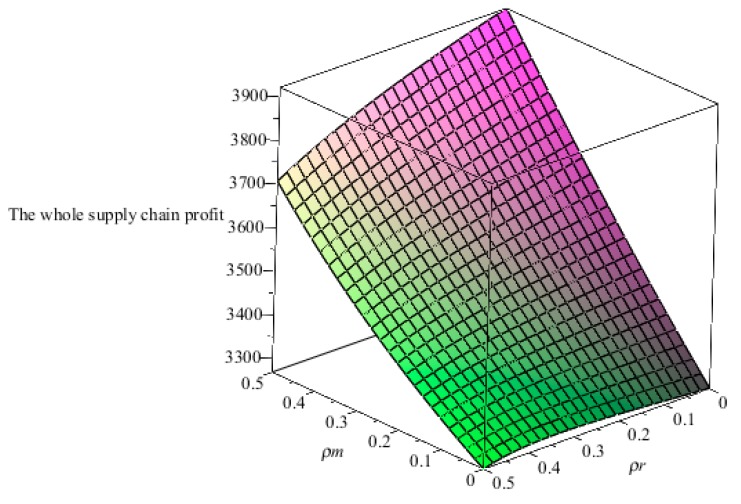
The relationship between the altruistic preference coefficients of two manufacturers and the retailer and the whole supply chain profit.

From Figure 8, the relationship between the altruistic preference coefficients of two manufacturers and the retailer and the whole supply chain profit, we can see that when two manufacturers have higher altruistic preference coefficients and the retailer has a lower altruistic preference coefficient, the whole supply chain profit is higher. When the retailer has a higher altruistic preference coefficient and two manufacturers have lower altruistic preference coefficients, the whole supply chain profit is lower.

### 5.4. Comparative Analysis

**Figure 9:**
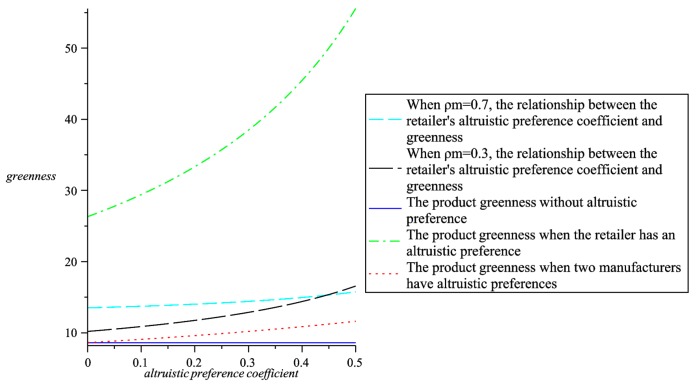
The relationship between product greenness and altruistic preference coefficients in four models

**Figure 10:**
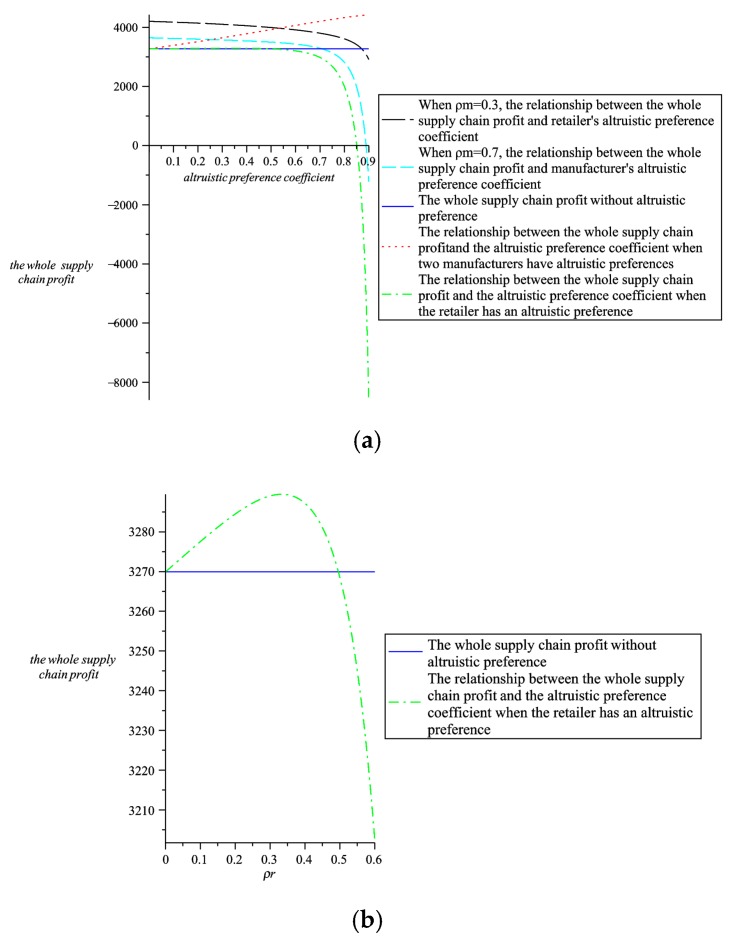
The relationship between the whole supply chain profit and altruistic preference coefficients. (**a**) The relationship between the whole supply chain profit and altruistic preference coefficients in four models. (**b**)The relationship between the whole supply chain profit and altruistic preference coefficients when the retailer has altruistic preferences and when there are no altruistic preferences.

**Figure 11:**
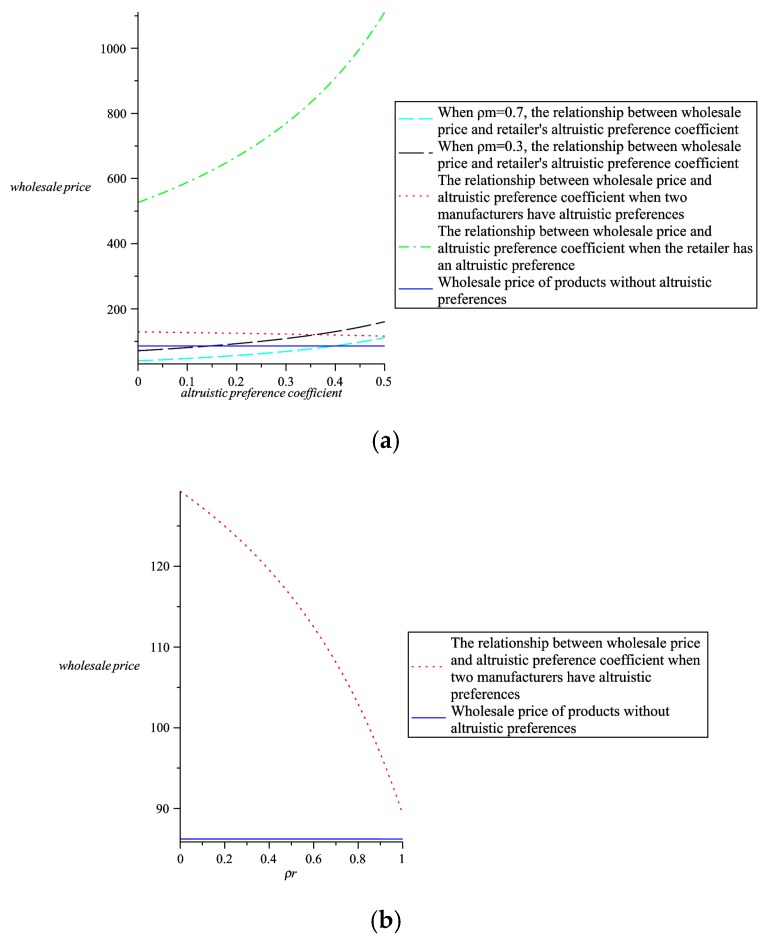
The relationship between wholesale prices and altruistic preference coefficients (**a**) The relationship between wholesale prices and altruistic preference coefficients in four models. (**b**) The relationship between wholesale prices and altruistic preference coefficients when two manufacturers have altruistic preferences.

**Figure 12:**
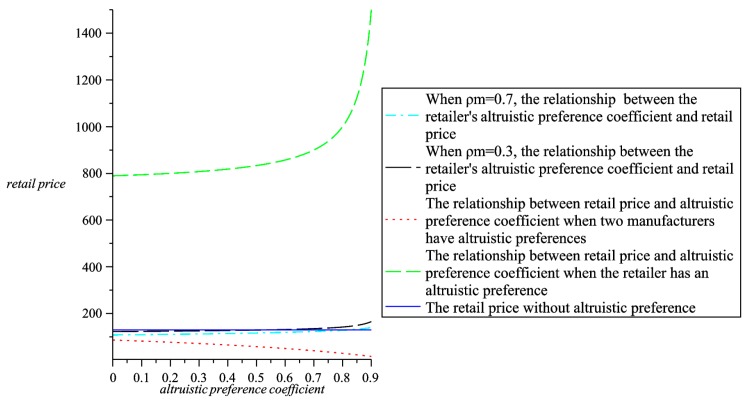
The relationship between retail prices and altruistic preference coefficients in four models.

Maintain the altruistic preference coefficient and analyze the relationship between greenness, wholesale prices, retail prices, the whole supply chain profit and altruistic preference coefficients. Through comparative analysis, further explain the choice of altruistic preference in four models. As shown in Figure 9, Figure 10, Figure 11 and Figure 12.

As can be seen from Figure 9, the product greenness when there are with altruistic preferences is higher than the product greenness when there are no altruistic preferences. At the same time, we also found that when the retailer has altruistic preference, the product has the highest greenness, and two manufacturers with the altruistic preferences have the lowest greenness. Therefore, from the perspective of product greenness, when the retailer has higher altruistic preferences, consumers can get the products with higher greenness.

It can be seen from Figure 10b that when the retailer’s altruistic preference is less than 0.5, the whole supply chain profit when the retailer has an altruistic preference is higher than when there are no altruistic preferences. It can be seen from Figure 10a that when the retailer’s altruistic preference coefficient is less than 0.5, the whole supply chain profit when there are with altruistic preferences is higher than when there are no altruistic preferences. At the same time, we can observe from Figure 10a that when two manufacturers and one retailer all have altruistic preferences and two manufacturers adopt higher altruistic preference coefficients and the retailer adopts a lower altruistic preference coefficient, the whole supply chain profit is higher than other three cases. Therefore, from the perspective of the whole supply chain profit, two manufacturers and one retailer all have altruistic preferences, and two manufacturers adopt higher altruistic preferences, and one retailer adopts a lower altruistic preference, the whole supply chain profit is the highest.

From Figure 11b we can see that when two manufacturers have altruistic preferences, the wholesale prices are higher than when there are no altruistic preferences. According to the analysis results in Section 5.2, in order to attract consumers and increase product sales, two manufacturers should adopt a higher altruistic preference coefficient. From Figure 11a we can observe that when two manufacturers and one retailer all have altruistic preferences and two manufacturer adopts a higher altruistic preference and the retailer adopts a lower altruistic preference, the wholesale prices are lower than the other three cases. Therefore, from the perspective of wholesale prices, two manufacturers and one retailer all have altruistic preferences, and two manufacturers adopt higher altruistic preferences, and one retailers adopt lower altruistic preferences, wholesale prices are the lowest.

As can be seen from Figure 12, in contrast to the other three cases, two manufacturers have the lowest retail prices when they have altruistic preferences, and are lower than the retail prices when there are no altruistic preferences. Comparing Figure 12 with Figure 11b, we find that when two manufacturers have altruistic preference coefficients, although the wholesale prices are higher than when there are no altruistic preferences, the retail prices are lower than when there is no altruistic preference. At the same time, as shown in Figure 10a, when two manufacturers have altruistic preference coefficients, the whole supply chain profit is higher than the whole supply chain profit when the retailer has an altruistic preference, which shows that from the perspective of pricing and the whole supply chain profit, two manufacturers can achieve better supply chain efficiency when they have altruistic preference compared with the retailer with altruistic preference. However, looking at Figure 9, it can be seen that the products has a lower greenness when two manufacturers have altruistic preferences than the other two models with altruistic preferences. In summary, through comparative analysis of numerical examples, we can see that:

(i) When two manufacturers and one retailer have altruistic preferences, the product greenness is higher than when there are no preferences.
(ii) When two manufacturers and one retailer have altruistic preferences, the whole supply chain profit is higher than when there are no preferences.
(iii) From the perspective of pricing and the whole supply chain profit, compared with the retailer with altruistic preference, two manufacturers can achieve better supply chain efficiency when they have altruistic preference, but the greenness is relatively low.
(iv) When two manufacturers and one retailer have altruistic preferences, two manufacturers should adopt higher altruistic preference coefficients, and the retailer should adopt a lower altruistic preference coefficient.

## 6. Conclusions

This paper establishes a three-stage supply chain consisting of two manufacturers and one retailer, considering the horizontal co-opetition relationship between two manufacturers is cooperation. Researching the Stackelberg game between two manufacturers and retailer, the manufacturers and the retailer make products greenness and pricing decisions when they have altruistic preferences. Through the model construction and numerical analysis, in the cooperative supply chain, when the retailer has altruistic preference, the retailer’s altruistic preference coefficient should not be too high for the retailer’s profit. When two manufacturers have altruistic preferences, the manufacturers will choose higher level of altruistic preference coefficients for the profit of the retailer. From the perspective of the whole supply chain profit, when two manufacturers and the retailer have altruistic preferences, two manufacturers adopt higher altruistic preference coefficients, while the retailer adopts a lower altruistic preference coefficient, the whole supply chain will receive the higher profit. Through analysis, in the three cases where the retailer has altruistic preferences, two manufacturers have altruistic preferences, and two manufacturers and one retailer all have altruistic preferences, two manufacturers should adopt higher altruistic preference coefficients, and the retailer should adopt a lower altruistic preference coefficient, and the product greenness under the three altruistic preferences is higher than the product greenness when there is no altruistic preferences.

## Figures and Tables

**Table 1:** The notations in the cooperative supply chain decision models.

**Parameters**	
*M_i_*	Manufacturer *i*, *i*∈(1,2)
*R*	Retailer
*α*	The potential market demand quantity
*β*	Price elasticity of demand
*θ*	The cross-price sensitivity coefficient
*τ*	The green sensitivity coefficient of consumers
Π*_mi_*	The profit of manufacturer *i*
Π*_M_*	The mutual profit of two manufacturers
Π*_R_*	The profit of retailer *R*
*U_mi_*	The altruistic utility value of manufacturer *i*
*U_M_*	The mutual altruistic utility value of two manufacturers
*U_R_*	The altruism utility value of retailer *R*
Π*_sc_*	The whole supply chain profit
**Decision Variables**	
*e_i_*	The product greenness produced by manufacturer *i*
*p_i_*	The retail price of the products produced by manufacturer *i*
*w_i_*	The wholesale price of manufacturer *i*
*ρ_m_*	The mutual altruistic preference coefficient of two manufacturers *M*_1_, *M*_2_
*ρ_r_*	The altruistic preference coefficient of the retailer
**Superscript/Subscripts**	
()*	Optimal results

## Appendix A

*Proof of Proposition 1*. When making greenness and pricing decisions, the Stackelberg game between two manufacturers and one retailer, and the order of the game is as follows: (1) *M*_1_ and *M*_2_ are the leaders. Two manufacturers make decisions of greenness and pricing according to mutual profit maximization principle of *M*_1_ and *M*_2_; (2) the retailer is the follower. Based on two manufacturers’ decisions, the retailer makes the wholesale prices according to the profit maximization principle.

Using the inverse derivation method, according to the Equation (1), the retailer’s profit function can be obtained, based on two manufacturers’ *w*_1_, *w*_2_, *e*_1_, *e*_2_. Solving the Hessian Matrix for Equation (2), the result is as follows:

(A1) $$H_{1}=\left[\begin{array}{cc} \frac{\partial^{2}\Pi_{R}}{\partial p_{1}^{2}} & \frac{\partial^{2}\Pi_{R}}{\partial p_{1}\partial p_{2}}\\ \frac{\partial^{2}\Pi_{R}}{\partial p_{2}\partial p_{2}} & \frac{\partial^{2}\Pi_{R}}{\partial p_{2}^{2}} \end{array}\right]=\left[\begin{array}{cc} -2\beta & 2\theta \\ 2\theta & -2\beta \end{array}\right]$$

If $\beta^{2}-\theta^{2}>0$, Hessian Matrix is negative definite, and the profit function of retailers $\Pi_{R}$ is strictly concave with respect to *p*_1_ and *p*_2_, which means that the retailer’s profit has a maximum value. Assuming $\frac{\partial \Pi_{R}}{\partial p_{i}}=0$, taking the partial derivatives of the retailer’s utility with respect to *p*_1_ and *p*_2_, we can obtain that:

(A2) $$\begin{array}{l} p_{1}=\frac{\alpha (\beta +\theta )+\tau (e_{1}\beta +e_{2}\theta )+w_{1}(\beta^{2}-\theta^{2})}{2(\beta^{2}-\theta^{2})}\\ p_{2}=\frac{\alpha (\beta +\theta )+\tau (e_{1}\theta +e_{2}\beta )+w_{1}(\beta^{2}-\theta^{2})}{2(\beta^{2}-\theta^{2})} \end{array}$$

Substituting Equation (A2) into the manufacturers’ mutual profit function Equation (1), and obtaining second-order partial derivatives of *w*_1_, *w*_2_, *e*_1_, *e*_2_ for function Equation (1), we can obtain the Hessian Matrix of the two manufacturers’ mutual profit function as follow:

(A3) $$H_{2}=\left[\begin{array}{cccc} \frac{\partial^{2}\Pi_{M}}{\partial w_{1}^{2}} & \frac{\partial^{2}\Pi_{M}}{\partial w_{1}\partial w_{2}} & \frac{\partial^{2}\Pi_{M}}{\partial w_{1}\partial e_{1}} & \frac{\partial^{2}\Pi_{M}}{\partial w_{1}\partial e_{2}}\\ \frac{\partial^{2}\Pi_{M}}{\partial w_{2}\partial w_{1}} & \frac{\partial^{2}\Pi_{M}}{\partial w_{2}^{2}} & \frac{\partial^{2}\Pi_{M}}{\partial w_{2}\partial e_{1}} & \frac{\partial^{2}\Pi_{M}}{\partial w_{2}\partial e_{2}}\\ \frac{\partial^{2}\Pi_{M}}{\partial e_{1}\partial w_{1}} & \frac{\partial^{2}\Pi_{M}}{\partial e_{1}\partial w_{2}} & \frac{\partial^{2}\Pi_{M}}{\partial e_{1}^{2}} & \frac{\partial^{2}\Pi_{M}}{\partial e_{1}\partial e_{2}}\\ \frac{\partial^{2}\Pi_{M}}{\partial e_{2}\partial w_{1}} & \frac{\partial^{2}\Pi_{M}}{\partial e_{2}\partial w_{2}} & \frac{\partial^{2}\Pi_{M}}{\partial e_{2}\partial e_{1}} & \frac{\partial^{2}\Pi_{M}}{\partial e_{2}^{2}} \end{array}\right]=\left[\begin{array}{cccc} -\beta & \theta & \frac{\tau }{2} & 0\\ \theta & -\beta & 0 & \frac{\tau }{2}\\ \frac{\tau }{2} & 0 & -1 & 0\\ 0 & \frac{\tau }{2} & 0 & -1 \end{array}\right]$$

When $\tau^{2}<4(\beta -\theta )$, Hessian Matrix is negative definite, which means that mutual profit function is concave function with respect to $w_{1},w_{2},e_{1},e_{2}$ and there is a maximum value. Taking the partial derivatives of the two manufacturers’ mutual profit $\Pi_{M}$ with respect to $w_{1},w_{2},e_{1},e_{2}$ and setting them equal to 0, the optimal results are as follows:

(A4) $$\begin{array}{l} e_{i}{}^{NNN*}=\frac{\alpha \tau }{4\beta -4\theta -\tau^{2}}\\ w_{i}{}^{NNN*}=\frac{2\alpha }{4\beta -4\theta -\tau^{2}} \end{array}$$

Substituting Equation (A4) into Equation (A2), the optimal retail prices are as follows:

(A5) $$p_{i}{}^{NNN*}=\frac{3\alpha }{4\beta -4\theta -\tau^{2}}$$

## Appendix B

*Proof of Proposition 2.* When making greenness and pricing decisions, the Stackelberg game between two manufacturers and one retailer, and the order of the game is as follows: (1) *M*_1_ and *M*_2_ are the leaders. The manufacturers make decisions of greenness and pricing according to mutual profit maximization principle of *M*_1_ and *M*_2_; (2) the retailer is the follower. Based on two manufacturers’ decisions, the retailer makes the wholesale prices according to the utility maximization principle.

Using the inverse derivation method, according to the Equation (1), the retailer’s utility function can be obtained, based on the manufacturers’ $w_{1},w_{2},e_{1},e_{2}$. Solving the Hessian Matrix for Equation (5), the result as is as follows:

(A6) $$H_{3}=\left[\begin{array}{cc} \frac{\partial^{2}U_{R}}{\partial P_{1}^{2}} & \frac{\partial^{2}U_{R}}{\partial P_{1}\partial P_{2}}\\ \frac{\partial^{2}U_{R}}{\partial P_{2}\partial P_{2}} & \frac{\partial^{2}U_{R}}{\partial P_{2}^{2}} \end{array}\right]=\left[\begin{array}{cc} -2\beta & 2\theta \\ 2\theta & -2\beta \end{array}\right]$$

If $\beta^{2}-\theta^{2}>0$, Hessian Matrix is negative definite, and the profit function of the retailer *U_R_* is strictly concave with respect to *p*_1_ and *p*_2_, which means that the retailer’s utility has a maximum value. Assuming $\frac{\partial U_{R}}{\partial p_{i}}=0$, taking the partial derivatives of the retailer’s utility with respect to *p*_1_ and *p*_2_, we can obtain that:

(A7) $$\begin{array}{l} p_{1}=\frac{\alpha (\beta +\theta )+\tau (e_{1}\beta +e_{2}\theta )+w_{1}(\beta^{2}-\theta^{2})}{2(\beta^{2}-\theta^{2})}\\ p_{2}=\frac{\alpha (\beta +\theta )+\tau (e_{1}\theta +e_{2}\beta )+w_{1}(\beta^{2}-\theta^{2})}{2(\beta^{2}-\theta^{2})} \end{array}$$

Substituting Equation (A7) into the manufacturers’ mutual profit function Equation (1), and obtaining second-order partial derivatives of $w_{1},w_{2},e_{1},e_{2}$ for function Equation (1), we can obtain the Hessian Matrix of the two manufacturers’ mutual profit function as follows:

(A8) $$H_{4}=\left[\begin{array}{cccc} \frac{\partial^{2}\Pi_{M}}{\partial w_{1}^{2}} & \frac{\partial^{2}\Pi_{M}}{\partial w_{1}\partial w_{2}} & \frac{\partial^{2}\Pi_{M}}{\partial w_{1}\partial e_{1}} & \frac{\partial^{2}\Pi_{M}}{\partial w_{1}\partial e_{2}}\\ \frac{\partial^{2}\Pi_{M}}{\partial w_{2}\partial w_{1}} & \frac{\partial^{2}\Pi_{M}}{\partial w_{2}^{2}} & \frac{\partial^{2}\Pi_{M}}{\partial w_{2}\partial e_{1}} & \frac{\partial^{2}\Pi_{M}}{\partial w_{2}\partial e_{2}}\\ \frac{\partial^{2}\Pi_{M}}{\partial e_{1}\partial w_{1}} & \frac{\partial^{2}\Pi_{M}}{\partial e_{1}\partial w_{2}} & \frac{\partial^{2}\Pi_{M}}{\partial e_{1}^{2}} & \frac{\partial^{2}\Pi_{M}}{\partial e_{1}\partial e_{2}}\\ \frac{\partial^{2}\Pi_{M}}{\partial e_{2}\partial w_{1}} & \frac{\partial^{2}\Pi_{M}}{\partial e_{2}\partial w_{2}} & \frac{\partial^{2}\Pi_{M}}{\partial e_{2}\partial e_{1}} & \frac{\partial^{2}\Pi_{M}}{\partial e_{2}^{2}} \end{array}\right]=\left[\begin{array}{cccc} -\beta (1-\rho_{r}) & \theta (1-\rho_{r}) & \frac{\tau }{2} & 0\\ \theta (1-\rho_{r}) & -\beta (1-\rho_{r}) & 0 & \frac{\tau }{2}\\ \frac{\tau }{2} & 0 & -1 & 0\\ 0 & \frac{\tau }{2} & 0 & -1 \end{array}\right]$$

When $\tau^{2}<4(\beta -\theta )$, Hessian Matrix is negative definite, which means that mutual profit function is concave function with respect to $w_{1},w_{2},e_{1},e_{2}$ and there is a maximum value. Taking the partial derivatives of the two manufacturers’ mutual profit $\Pi_{M}$ with respect to $w_{1},w_{2},e_{1},e_{2}$ and setting them equal to 0, the optimal results are as follows:

(A9) $$\begin{array}{l} e_{i}^{NNR*}=\frac{\alpha \tau }{4\beta -4\theta -\tau^{2}+4\rho_{r}(\theta -\beta )}\\ w_{i}^{NNR*}=\frac{2\alpha }{4\beta -4\theta -\tau^{2}+4\rho_{r}(\theta -\beta )} \end{array}$$

Substituting Equation (A9) into Equation (A7), the optimal retail prices are as follows:

(A10) $$P_{i}^{NNR*}=\frac{3\alpha (1-\rho_{r})}{4\beta -4\theta -\tau^{2}+4\rho_{r}(\theta -\beta )}$$

## Appendix C

*Proof of Proposition 3.* The retailer will only take altruistic preference when it is profitable. If the retailer has harmed his own profit after taking the altruistic preference, from a rational point of view, the retailer will abandon the altruistic preference, but choose self-interested behavior. From the above conclusions, the following relationship can be obtained:

(A11) $$\Pi_{R}^{NNN*}-\Pi_{{}_{R}}^{NNR*}=\frac{2\alpha^{2}(\beta -\theta )}{{\left(\tau^{2}-4\beta +4\theta \right)}^{2}}-\frac{6(\beta -\theta )\alpha^{2}(\rho_{r}-1)(\rho_{r}-\frac{1}{3})}{{\left[4(\beta -\theta )\rho_{r}+\tau^{2}-4\beta +4\theta \right]}^{2}}>0$$

(A12) $$\Pi_{M}^{NNR*}-\Pi_{M}^{NNN*}=\frac{\alpha^{2}}{4\beta -4\theta -\tau^{2}-4\rho_{r}(\beta -\theta )}-\frac{\alpha^{2}}{4\beta -4\theta -\tau^{2}}>0$$

It can be seen from the above relationship that after the retailer adopts the altruistic preference behavior, its own profit is less than the profit when taking the self-interested behavior. The retailer knows that after taking the altruistic preference behavior, its own profits will decline, but still take altruistic preference behavior, indicating that the retailer is still profitable after taking altruistic preferences. We can obtain that:

(A13) $$P_{1}^{NNR*}+P_{2}^{NNR*}-w_{1}^{NNR*}-w_{2}^{NNR*}=\frac{2\alpha (1-3\rho_{r})}{4\beta -4\theta -\tau^{2}+4\rho_{r}(\theta -\beta )}>0,0<\rho_{r}<\frac{1}{3}$$

## Appendix D

*Proof of Proposition 4.*

(A14) $$\begin{array}{l} \frac{\partial e_{i}^{NNR*}}{\partial \rho_{r}}=\frac{4\alpha \tau (\beta -\theta )}{4\beta -4\theta -\tau^{2}-4\rho_{r}(\beta -\theta )}>0\\ \frac{\partial w_{i}^{NNR*}}{\partial \rho_{r}}=\frac{4\alpha (\beta -\theta )}{{\left[4\beta -4\theta -\tau^{2}-4\rho_{r}(\beta -\theta )\right]}^{2}}>0\\ \frac{\partial p_{i}^{NNR*}}{\partial \rho_{r}}=\frac{3\alpha \tau^{2}}{{\left[4\beta -4\theta -\tau^{2}-4\rho_{r}(\beta -\theta )\right]}^{2}}>0 \end{array}$$

## Appendix E

*Proof of Proposition 5.* When making greenness and pricing decisions, the Stackelberg game between two manufacturers and one retailer, and the order of the game is as follows: (1) *M*_1_ and *M*_2_ are the leaders. The manufacturers make decisions of greenness and pricing according to mutual utility maximization principle of *M*_1_ and *M*_2_; (2) the retailer is the follower. Based on two manufacturers’ decisions, the retailer makes the wholesale prices according to the interest maximization principle.

Using the inverse derivation method, according to the Equation (8), the retailer’s profit function can be obtained, based on two manufacturers’$w_{1},w_{2},e_{1},e_{2}$. Solving the Hessian Matrix for Equation (2), the result as is as follows:

(A15) $$H_{5}=\left[\begin{array}{cc} \frac{\partial^{2}\Pi_{R}}{\partial P_{1}^{2}} & \frac{\partial^{2}\Pi_{R}}{\partial P_{1}\partial P_{2}}\\ \frac{\partial^{2}\Pi_{R}}{\partial P_{2}\partial P_{2}} & \frac{\partial^{2}\Pi_{R}}{\partial P_{2}^{2}} \end{array}\right]=\left[\begin{array}{cc} -2\beta & 2\theta \\ 2\theta & -2\beta \end{array}\right]$$

If $\beta^{2}-\theta^{2}>0$, Hessian Matrix is negative definite, and the profit function of retailers Π*_R_* is strictly concave with respect to *p*_1_ and *p*_2_, which means that the retailer’s profit value has a maximum value. Assuming $\frac{\partial U_{R}}{\partial p_{i}}=0$, taking the partial derivatives of the retailer’s profit with respect to *p*_1_ and *p*_2_, we can obtain that:

(A16) $$\begin{array}{l} p_{1}=\frac{\alpha (\beta +\theta )+\tau (e_{1}\beta +e_{2}\theta )+w_{1}(\beta^{2}-\theta^{2})}{2(\beta^{2}-\theta^{2})}\\ p_{2}=\frac{\alpha (\beta +\theta )+\tau (e_{1}\theta +e_{2}\beta )+w_{1}(\beta^{2}-\theta^{2})}{2(\beta^{2}-\theta^{2})} \end{array}$$

Substituting Equation (A16) into the manufacturers’ mutual utility function Equation (8), and obtaining second-order partial derivatives of $w_{1},w_{2},e_{1},e_{2}$ for function Equation (8), respectively, we can obtain the Hessian Matrix of the two manufacturers’ mutual utility function, as follow:

(A17) $$\begin{array}{l} H_{6}=\left[\begin{array}{cccc} \frac{\partial^{2}U_{M}}{\partial w_{1}^{2}} & \frac{\partial^{2}U_{M}}{\partial w_{1}\partial w_{2}} & \frac{\partial^{2}U_{M}}{\partial w_{1}\partial e_{1}} & \frac{\partial^{2}U_{M}}{\partial w_{1}\partial e_{2}}\\ \frac{\partial^{2}U_{M}}{\partial w_{2}\partial w_{1}} & \frac{\partial^{2}U_{M}}{\partial w_{2}^{2}} & \frac{\partial^{2}U_{M}}{\partial w_{2}\partial e_{1}} & \frac{\partial^{2}U_{M}}{\partial w_{2}\partial e_{2}}\\ \frac{\partial^{2}U_{M}}{\partial e_{1}\partial w_{1}} & \frac{\partial^{2}U_{M}}{\partial e_{1}\partial w_{2}} & \frac{\partial^{2}U_{M}}{\partial e_{1}^{2}} & \frac{\partial^{2}U_{M}}{\partial e_{1}\partial e_{2}}\\ \frac{\partial^{2}U_{M}}{\partial e_{2}\partial w_{1}} & \frac{\partial^{2}U_{M}}{\partial e_{2}\partial w_{2}} & \frac{\partial^{2}U_{M}}{\partial e_{2}\partial e_{1}} & \frac{\partial^{2}U_{M}}{\partial e_{2}^{2}} \end{array}\right]\\ =\left[\begin{array}{cccc} -\beta +\frac{\beta \rho_{m}}{2} & \theta -\frac{\theta \rho_{m}}{2} & \frac{\tau (1-\rho_{m})}{2} & 0\\ \theta -\frac{\theta \rho_{m}}{2} & -\beta +\frac{\beta \rho_{m}}{2} & 0 & \frac{\tau (1-\rho_{m})}{2}\\ \frac{\tau (1-\rho_{m})}{2} & 0 & -1+\frac{\beta \tau^{2}\rho_{m}}{2(\beta^{2}-\theta^{2})} & \frac{\theta \tau^{2}\rho_{m}}{2(\beta^{2}-\theta^{2})}\\ 0 & \frac{\tau }{2} & \frac{\theta \tau^{2}\rho_{m}}{2(\beta^{2}-\theta^{2})} & -1+\frac{\beta \tau^{2}\rho_{m}}{2(\beta^{2}-\theta^{2})} \end{array}\right] \end{array}$$

When:

(A18) $$\begin{array}{l} (\rho_{m}{}^{2}-2\rho_{m}+\frac{1}{2})\beta \tau^{2}+(2-\rho_{m})(\beta^{2}-\theta^{2})<0\\ \tau^{4}\left[2\beta^{2}\rho_{m}(\rho_{m}-2)+\beta^{2}-\theta^{2}\right]+\tau^{2}\left[4\beta^{2}(1-\rho_{m})+\rho_{m}{}^{2}(\beta^{2}-\theta^{2})+4\theta (\beta -\theta )\right]-4{\left(\rho_{m}-2\right)}^{2}{\left(\beta^{2}-\theta^{2}\right)}^{2}>0 \end{array}$$

Hessian Matrix is negative definite, which means that mutual utility function is concave function with respect to $w_{1},w_{2},e_{1},e_{2}$ and there is a maximum value. Taking the partial derivatives of the two manufacturers’ mutual utility *U*_M_ with respect to $w_{1},w_{2},e_{1},e_{2}$ and setting them equal to 0, the optimal results are as the follows:

(A19) $$\begin{array}{l} e_{i}^{MMN*}=\frac{\alpha \tau }{4\beta -4\theta -\tau^{2}-2\rho_{m}(\beta -\theta )}\\ w_{i}^{MMN*}=\frac{2\alpha (1-\rho_{m})}{4\beta -4\theta -\tau^{2}-2\rho_{m}(\beta -\theta )} \end{array}$$

Substituting Equation (A19) into Equation (A16), the optimal retail prices are as follows:

(A20) $$p_{i}^{MMN*}=\frac{\alpha (3-2\rho_{m})}{4\beta -4\theta -\tau^{2}-2\rho_{m}(\beta -\theta )}$$

## Appendix F

*Proof of Proposition 6.* The following relation can be obtained from the above the conclusion:

(A21) $$\begin{array}{l} \frac{\partial e_{i}^{MMN*}}{\partial \rho_{m}}=\frac{2\tau \alpha (\beta -\theta )}{{\left[4\beta -4\theta -\tau^{2}-2\rho_{m}(\beta -\theta )\right]}^{2}}>0\\ \frac{\partial w_{i}^{MMN*}}{\partial \rho_{m}}=\frac{2\alpha (\tau^{2}-\beta +\theta )}{{\left[4\beta -4\theta -\tau^{2}-2\rho_{m}(\beta -\theta )\right]}^{2}}<0\\ \frac{\partial p_{i}^{MMN*}}{\partial \rho_{m}}=\frac{2\alpha (\tau^{2}-2\beta +2\theta )}{{\left[4\beta -4\theta -\tau^{2}-2\rho_{m}(\beta -\theta )\right]}^{2}}<0 \end{array}$$

## Appendix G

*Proof of Proposition 7.* From the results of the product greenness when *M*_1_ and *M*_2_ have both altruistic preferences and the retailer has altruistic preference, we can see that their molecules of the product greenness are the same, and their relationship can be known only by comparing the denominators:

(A22) $$e_{i}^{MMN*}-e_{i}^{NNR*}=\frac{\alpha \tau }{4\beta -4\theta -\tau^{2}-2\rho_{m}(\beta -\theta )}-\frac{\alpha \tau }{4\beta -4\theta -\tau^{2}-4\rho_{r}(\beta -\theta )}$$

When $\rho_{m}=2\rho_{r}$,$e_{m}^{MMN*}=e_{m}^{NNR*}$; when $\rho_{m}>2\rho_{r}$,$e_{m}^{MMN*}>e_{m}^{NNR*}$; when $\rho_{m}<2\rho_{r}$,$e_{m}^{MMN*}<e_{m}^{NNR*}$.

Then compared with the product greenness when two manufacturers and one retailer are full self-interest:

(A23) $$\begin{array}{l} e_{i}^{MMN*}-e_{i}^{NNN*}=\frac{\alpha \tau }{4\beta -4\theta -\tau^{2}-4\rho_{r}(\beta -\theta )}-\frac{\alpha \tau }{4\beta -4\theta -\tau^{2}}>0\\ e_{i}^{NNR*}-e_{i}^{NNN*}=\frac{\alpha \tau }{4\beta -4\theta -\tau^{2}-2\rho_{m}(\beta -\theta )}-\frac{\alpha \tau }{4\beta -4\theta -\tau^{2}}>0 \end{array}$$

We can obtain that: $e_{i}^{MMN*}>e_{i}^{NNN*}$, $e_{i}^{NNR*}>e_{i}^{NNN*}$.

## Appendix H

*Proof of Proposition 8.* According to the condition of Hessian Matrix $H_{6}$ with negative definite, when $(\rho_{m}{}^{2}-2\rho_{m}+\frac{1}{2})\beta \tau^{2}+(2-\rho_{m})(\beta^{2}-\theta^{2})<0$, we can derive that $\rho_{m}{}^{2}-2\rho_{m}+\frac{1}{2}<0$ is must be established, and we can obtain $1-\sqrt{\frac{1}{2}}<\rho_{m}<1$.

## Appendix I

*Proof of Proposition 9.* When making greenness and pricing decisions, the Stackelberg game between two manufacturers and one retailer, and the order of the game is as follows: (1) *M*_1_ and *M*_2_ are the leaders. The manufacturers make decisions of greenness and pricing according to mutual utility maximization principle of *M*_1_ and *M*_2_; (2) the retailer is the follower. Based on two manufacturers’ decisions, the retailer makes the wholesale prices according to the utility maximization principle.

Using the inverse derivation method, according to Equation (12), the retailer’s utility function can be obtained, based on the manufacturers’$w_{1},w_{2},e_{1},e_{2}$. Solving the Hessian Matrix for Equation (13), the result as is as follows:

(A24) $$H_{7}=\left[\begin{array}{cc} \frac{\partial^{2}U_{R}}{\partial P_{1}^{2}} & \frac{\partial^{2}U_{R}}{\partial P_{1}\partial P_{2}}\\ \frac{\partial^{2}U_{R}}{\partial P_{2}\partial P_{2}} & \frac{\partial^{2}U_{R}}{\partial P_{2}^{2}} \end{array}\right]=\left[\begin{array}{cc} -2\beta & 2\theta \\ 2\theta & -2\beta \end{array}\right]$$

If $\beta^{2}-\theta^{2}>0$, Hessian Matrix is negative definite, and the utility function of retailers $U_{R}$ is strictly concave with respect to *p*_1_ and *p*_2_, which means that the retailer’s utility value has a maximum value. Assuming $\frac{\partial U_{R}}{\partial p_{i}}=0$, taking the partial derivatives of the retailer’s profit with respect to *p*_1_ and *p*_2_, we can obtain that:

(A25) $$\begin{array}{l} p_{1}=\frac{\alpha (\beta +\theta )+\tau (e_{1}\beta +e_{2}\theta )+w_{1}(\beta^{2}-\theta^{2})}{2(\beta^{2}-\theta^{2})}\\ p_{2}=\frac{\alpha (\beta +\theta )+\tau (e_{1}\theta +e_{2}\beta )+w_{1}(\beta^{2}-\theta^{2})}{2(\beta^{2}-\theta^{2})} \end{array}$$

Substituting Equation (A25) into the manufacturers’ mutual profit function Equation (12), and then obtaining second-order partial derivatives of $w_{1},w_{2},e_{1},e_{2}$ for function Equation (12), respectively, we can obtain the Hessian Matrix of the two manufacturers’ mutual utility function, as follows:

(A26) $$\begin{array}{l} H_{8}=\left[\begin{array}{cccc} \frac{\partial^{2}U_{M}}{\partial w_{1}^{2}} & \frac{\partial^{2}U_{M}}{\partial w_{1}\partial w_{2}} & \frac{\partial^{2}U_{M}}{\partial w_{1}\partial e_{1}} & \frac{\partial^{2}U_{M}}{\partial w_{1}\partial e_{2}}\\ \frac{\partial^{2}U_{M}}{\partial w_{2}\partial w_{1}} & \frac{\partial^{2}U_{M}}{\partial w_{2}^{2}} & \frac{\partial^{2}U_{M}}{\partial w_{2}\partial e_{1}} & \frac{\partial^{2}U_{M}}{\partial w_{2}\partial e_{2}}\\ \frac{\partial^{2}U_{M}}{\partial e_{1}\partial w_{1}} & \frac{\partial^{2}U_{M}}{\partial e_{1}\partial w_{2}} & \frac{\partial^{2}U_{M}}{\partial e_{1}^{2}} & \frac{\partial^{2}U_{M}}{\partial e_{1}\partial e_{2}}\\ \frac{\partial^{2}U_{M}}{\partial e_{2}\partial w_{1}} & \frac{\partial^{2}U_{M}}{\partial e_{2}\partial w_{2}} & \frac{\partial^{2}U_{M}}{\partial e_{2}\partial e_{1}} & \frac{\partial^{2}U_{M}}{\partial e_{2}^{2}} \end{array}\right]\\ =\left[\begin{array}{cccc} \frac{\beta (1-\rho_{r})(-2+\rho_{m}+\rho_{m}\rho_{r})}{2} & \frac{\theta (1-\rho_{r})(-2+\rho_{m}+\rho_{m}\rho_{r})}{2} & \frac{\tau (1-\rho_{m})}{2} & 0\\ \frac{\theta (1-\rho_{r})(-2+\rho_{m}+\rho_{m}\rho_{r})}{2} & \frac{\beta (1-\rho_{r})(-2+\rho_{m}+\rho_{m}\rho_{r})}{2} & 0 & \frac{\tau (1-\rho_{m})}{2}\\ \frac{\tau (1-\rho_{m})}{2} & 0 & -1+\frac{\rho_{m}\beta \tau^{2}}{2(\beta^{2}-\theta^{2})} & \frac{\rho_{m}\theta \tau^{2}}{2(\beta^{2}-\theta^{2})}\\ 0 & \frac{\tau (1-\rho_{m})}{2} & \frac{\rho_{m}\theta \tau^{2}}{2(\beta^{2}-\theta^{2})} & -1+\frac{\rho_{m}\beta \tau^{2}}{2(\beta^{2}-\theta^{2})} \end{array}\right] \end{array}$$

When ${\left(\rho_{m}\rho_{r}-1\right)}^{2}\tau^{2}+2(\beta +\theta )\left[\rho_{m}(1+\rho_{r})-2\right](1-\rho_{r})<0$, the Hessian Matrix is negative definite, which means that the mutual utility function is a concave function with respect to $w_{1},w_{2},e_{1},e_{2}$ and there is a maximum value. Taking the partial derivatives of the two manufacturers’ mutual utility $U_{M}$ with respect to $w_{1},w_{2},e_{1},e_{2}$ and setting them equal to 0, the optimal results are as follows:

(A27) $$\begin{array}{l} e_{i}^{MMR*}=-\frac{{\left(\rho_{m}\rho_{r}-1\right)}^{2}\tau \alpha }{{\left(\rho_{m}\rho_{r}-1\right)}^{2}\tau^{2}-2(\beta -\theta )\left[\rho_{m}(\rho_{r}{}^{2}-1)+2(1-\rho_{r})\right]}\\ w_{i}^{MMR*}=-\frac{2(\rho_{m}-1)\alpha }{{\left(\rho_{m}\rho_{r}-1\right)}^{2}\tau^{2}-2(\beta -\theta )\left[\rho_{m}(\rho_{r}{}^{2}-1)+2(1-\rho_{r})\right]} \end{array}$$

Substituting Equation (A27) into Equation(A25), we can obtain that:

(A28) $$p_{i}^{MMR*}=-\frac{(\rho_{r}-1)\left[-3+(\rho_{r}+2)\rho_{m}\right]\alpha }{{\left(\rho_{m}\rho_{r}-1\right)}^{2}\tau^{2}-2(\beta -\theta )\left[\rho_{m}(\rho_{r}{}^{2}-1)+2(1-\rho_{r})\right]}$$

## Appendix J

*Proof of Proposition 10.* From the above the conclusion, the following relationship can be obtained:

(A29) $$\frac{\partial e_{i}^{*}}{\partial \rho_{r}}=\frac{-4(\rho_{m}\rho_{r}-1)\tau \alpha {\left(\rho_{m}-1\right)}^{2}(\beta -\theta )}{{\left\{\left[\rho_{m}(\rho_{m}\tau^{2}+2\theta -2\beta )\right]\rho_{r}{}^{2}+2(2\beta -2\theta -\rho_{m}\tau^{2})\rho_{r}+(2\beta -2\theta )\rho_{m}+4\theta -4\beta +\tau^{2}\right\}}^{2}}>0$$

(A30) $$\frac{\partial p_{i}^{*}}{\partial \rho_{m}}=\frac{\left\{\left[\rho_{r}(\rho_{m}\rho_{r}-1)(\rho_{m}\rho_{r}-5)+2\rho_{m}^{2}\rho_{r}^{2}\right]\tau^{2}+(\beta -\theta ){\left(\rho_{r}-1\right)}^{2}\right\}\alpha (\rho_{r}-1)}{{\left\{\left[\rho_{m}(\rho_{m}\tau^{2}+2\theta -2\beta )\right]\rho_{r}{}^{2}+2(2\beta -2\theta -\rho_{m}\tau^{2})\rho_{r}+(2\beta -2\theta )\rho_{m}+4\theta -4\beta +\tau^{2}\right\}}^{2}}<0$$

(A31) $$\frac{\partial w_{i}^{*}}{\partial \rho_{m}}=\frac{-2\alpha \left\{{\left(\rho_{m}\rho_{r}-1\right)}^{2}+2\rho_{r}\left[\rho_{m}(1-\rho_{r})+1\right]+2(\beta -\theta ){\left(\rho_{r}-1\right)}^{2}\right\}}{{\left\{\left[\rho_{m}(\rho_{m}\tau^{2}+2\theta -2\beta )\right]\rho_{r}{}^{2}+2(2\beta -2\theta -\rho_{m}\tau^{2})\rho_{r}+(2\beta -2\theta )\rho_{m}+4\theta -4\beta +\tau^{2}\right\}}^{2}}<0$$

## Appendix K

*Proof of Proposition 11.* From the results of the wholesale prices when two manufacturers have altruistic preferences and two manufacturers and one retailer all have altruistic preferences, we can see that their molecules of the wholesale prices are the same, so their relationship can be known only by comparing the denominators:

(A32) $$\begin{array}{l} w_{i}^{MMR*}-w_{i}^{MMN*}=\\ -\frac{2(\rho_{m}-1)\alpha }{{\left(\rho_{m}\rho_{r}-1\right)}^{2}\tau^{2}-2(\beta -\theta )\left[\rho_{m}(\rho_{r}{}^{2}-1)+2(1-\rho_{r})\right]}-\frac{2(\rho_{m}-1)\alpha }{4\beta -4\theta -\tau^{2}-2\rho_{m}(\beta -\theta )} \end{array}$$

We can get the relationship between the denominators of the wholesale prices between the two altruistic preference models.

(A33) $$\begin{array}{l} {\left(\rho_{m}\rho_{r}-1\right)}^{2}\tau^{2}-2(\beta -\theta )\left[\rho_{m}(\rho_{r}{}^{2}-1)+2(1-\rho_{r})\right]-(\tau^{2}+2\rho_{m}(\beta -\theta )-4\beta +4\theta )\\ =\left[\rho_{m}\tau^{2}+2(\theta -\beta )\right](\rho_{m}\rho_{r}-2)\rho_{r} \end{array}$$

When $\rho_{m}=\frac{2(\beta -\theta )}{\tau^{2}}$, $w_{i}^{MMR*}=w_{i}^{MMN*}$. When $\rho_{m}>\frac{2(\beta -\theta )}{\tau^{2}}$, $w_{i}^{MMR*}<w_{i}^{MMN*}$. When $\rho_{m}<\frac{2(\beta -\theta )}{\tau^{2}}$, $w_{i}^{MMR*}>w_{i}^{MMN*}$.

## Appendix L

*Proof of Proposition 12.* From the above the conclusion, we can be obtained that:

(A34) $$\begin{array}{l} e_{i}^{MMR*}-e_{i}^{MMN*}=-\frac{{\left(\rho_{m}\rho_{r}-1\right)}^{2}\tau \alpha }{{\left(\rho_{m}\rho_{r}-1\right)}^{2}\tau^{2}-2(\beta -\theta )\left[\rho_{m}(\rho_{r}{}^{2}-1)+2(1-\rho_{r})\right]}-\frac{\alpha \tau }{4\beta -4\theta -\tau^{2}-2\rho_{m}(\beta -\theta )}\\ =-\frac{2\alpha \tau \rho_{r}{\left(\rho_{m}-1\right)}^{2}(\rho_{m}\rho_{r}-2)(\beta -\theta )}{\left\{{\left(\rho_{m}\rho_{r}-1\right)}^{2}\tau^{2}-2(\beta -\theta )\left[\rho_{m}(\rho_{r}{}^{2}-1)+2(1-\rho_{r})\right]\}\{\tau^{2}-4\beta +4\theta +4\rho_{r}(\beta -\theta )\right\}}>0 \end{array}$$

We can derive that: $e_{i}^{MMR*}>e_{i}^{MMN*}>e_{i}^{NNN*}$.

## Appendix M

*Proof of Situation 1* **.**

(A35) $$\begin{array}{l} e_{i}^{MMR*}-e_{i}^{NNR*}=-\frac{{\left(\rho_{m}\rho_{r}-1\right)}^{2}\tau \alpha }{{\left(\rho_{m}\rho_{r}-1\right)}^{2}\tau^{2}-2(\beta -\theta )\left[\rho_{m}(\rho_{r}{}^{2}-1)+2(1-\rho_{r})\right]}-\frac{\alpha \tau }{4\beta -4\theta -\tau^{2}+4\rho_{r}(\theta -\beta )}\\ =-\frac{4\alpha \tau \rho_{m}(\beta -\theta )(\rho_{m}\rho_{r}{}^{2}-\frac{3}{2}\rho_{r}+\frac{1}{2})}{\left\{{\left(\rho_{m}\rho_{r}-1\right)}^{2}\tau^{2}-2(\beta -\theta )\left[\rho_{m}(\rho_{r}{}^{2}-1)+2(1-\rho_{r})\right]\}\{\tau^{2}-4\beta +4\theta +4\rho_{r}(\beta -\theta )\right\}} \end{array}$$

## Appendix N

*Proof of Situation 2.*

(A36) $$\frac{\partial e_{i}^{*}}{\partial \rho_{m}}=\frac{2(\rho_{r}-1)\left[\rho_{m}\rho_{r}{}^{2}+(\rho_{m}-3)\rho_{r}+1\right](\beta -\theta )\tau (\rho_{m}\rho_{r}-1)\alpha }{{\left\{\left[\rho_{m}(\rho_{m}\tau^{2}+2\theta -2\beta )\right]\rho_{r}{}^{2}+2(2\beta -2\theta -\rho_{m}\tau^{2})\rho_{r}+(2\beta -2\theta )\rho_{m}+4\theta -4\beta +\tau^{2}\right\}}^{2}}$$

## Appendix O

*Proof of Situation 3.*

(A37) $$\frac{\partial w_{i}^{*}}{\partial \rho_{r}}=\frac{-4\alpha (\rho_{m}\rho_{r}-1)(\rho_{m}-1)(\rho_{m}\tau^{2}+2\theta -2\beta )}{{\left\{\left[\rho_{m}(\rho_{m}\tau^{2}+2\theta -2\beta )\right]\rho_{r}{}^{2}+2(2\beta -2\theta -\rho_{m}\tau^{2})\rho_{r}+(2\beta -2\theta )\rho_{m}+4\theta -4\beta +\tau^{2}\right\}}^{2}}$$

## Appendix P

*Proof of Situation 4.*

(A38) $$\frac{\partial p_{i}^{*}}{\partial \rho_{r}}=\frac{\left[(\rho_{m}\rho_{r}-1)(\rho_{m}\rho_{r}+3)+4(1-\rho_{m}\rho_{r})\rho_{m}-2\rho_{m}(\beta -\theta ){\left(\rho_{r}-1\right)}^{2}\right]\alpha (\rho_{m}-1)}{{\left\{\left[\rho_{m}(\rho_{m}\tau^{2}+2\theta -2\beta )\right]\rho_{r}{}^{2}+2(2\beta -2\theta -\rho_{m}\tau^{2})\rho_{r}+(2\beta -2\theta )\rho_{m}+4\theta -4\beta +\tau^{2}\right\}}^{2}}$$
